# Photoacoustic imaging for cutaneous melanoma assessment: a comprehensive review

**DOI:** 10.1117/1.JBO.29.S1.S11518

**Published:** 2024-01-12

**Authors:** Joseph W. Fakhoury, Juliana Benavides Lara, Rayyan Manwar, Mohsin Zafar, Qiuyun Xu, Ricardo Engel, Maria M. Tsoukas, Steven Daveluy, Darius Mehregan, Kamran Avanaki

**Affiliations:** a Wayne State University School of Medicine, Detroit, Michigan, United States; b University of Illinois at Chicago, Richard and Loan Hill Department of Bioengineering, Chicago, Illinois, United States; c Wayne State University, Department of Biomedical Engineering, Detroit, Michigan, United States; d University of Illinois at Chicago, Department of Dermatology, Chicago, Illinois, United States; e Wayne State University School of Medicine, Department of Dermatology, Detroit, Michigan, United States

**Keywords:** photoacoustic imaging, photoacoustic microscopy, photoacoustic tomography, cutaneous melanoma, skin cancer, contrast agents

## Abstract

**Significance:**

Cutaneous melanoma (CM) has a high morbidity and mortality rate, but it can be cured if the primary lesion is detected and treated at an early stage. Imaging techniques such as photoacoustic (PA) imaging (PAI) have been studied and implemented to aid in the detection and diagnosis of CM.

**Aim:**

Provide an overview of different PAI systems and applications for the study of CM, including the determination of tumor depth/thickness, cancer-related angiogenesis, metastases to lymph nodes, circulating tumor cells (CTCs), virtual histology, and studies using exogenous contrast agents.

**Approach:**

A systematic review and classification of different PAI configurations was conducted based on their specific applications for melanoma detection. This review encompasses animal and preclinical studies, offering insights into the future potential of PAI in melanoma diagnosis in the clinic.

**Results:**

PAI holds great clinical potential as a noninvasive technique for melanoma detection and disease management. PA microscopy has predominantly been used to image and study angiogenesis surrounding tumors and provide information on tumor characteristics. Additionally, PA tomography, with its increased penetration depth, has demonstrated its ability to assess melanoma thickness. Both modalities have shown promise in detecting metastases to lymph nodes and CTCs, and an all-optical implementation has been developed to perform virtual histology analyses. Animal and human studies have successfully shown the capability of PAI to detect, visualize, classify, and stage CM.

**Conclusions:**

PAI is a promising technique for assessing the status of the skin without a surgical procedure. The capability of the modality to image microvasculature, visualize tumor boundaries, detect metastases in lymph nodes, perform fast and label-free histology, and identify CTCs could aid in the early diagnosis and classification of CM, including determination of metastatic status. In addition, it could be useful for monitoring treatment efficacy noninvasively.

## 1 Introduction

Over the last few decades, the global incidence of cutaneous melanoma (CM) has continued to rise.^1^^–^^5^ Melanoma is the fifth most common cancer in the United States, with high morbidity and mortality.^6^ While only 2% of skin cancers are diagnosed as melanoma, it accounts for 75% of skin cancer deaths annually.^7^^–^^10^ Melanoma tumor depth is an important prognostic factor, along with ulceration status.^11^ Early detection and diagnosis is critical.^12^ The 5-year survival rate is 99% for localized melanomas but decreases to 63% with regional metastases and 20% with distant metastases.^8^

Biopsies have long been considered the diagnostic standard for melanoma.^13^^,^^14^ Performing biopsies can result in pain, anxiety, scarring, and disfigurement for patients, as well as a considerable cost to the healthcare system. Given the high mortality associated with melanoma depth and metastasis, there is an urgent need for accurate, noninvasive methods to detect and monitor the disease. As a result, several noninvasive imaging techniques have been developed for skin imaging^15^^–^^17^ including, among others, infrared imaging, hyperspectral/multispectral imaging, reflectance confocal microscopy (RCM), optical coherence tomography (OCT),^18^^–^^35^ and photoacoustic (PA) imaging (PAI).^36^^–^^40^ Many noninvasive imaging modalities have been used to study different aspects of CM, as comprehensively described in review articles.^41^^–^^43^ Beyond their capabilities for an initial diagnosis, other opportunities for melanoma staging through imaging involve analysis of lymph nodes for metastases and detection of circulating (melanoma) tumor cells (CTCs).

PAI, also known as optoacoustic imaging, is an emerging noninvasive imaging modality^44^^–^^52^ in which a nano-pulsed excitation light is absorbed by tissue chromophores (such as hemoglobin, lipids, bilirubin, and melanin) or exogenous contrast agents (such as organic dyes or nanoparticles) leading to a transient localized thermoelastic expansion.^51^^,^^53^^–^^58^ This induces the generation of PA signals, which are then detected by an ultrasound (US) transducer and reconstructed into a two-dimensional (2D) or three-dimensional (3D) image. The strength of the PA signals correlates with tissue chromophore absorption properties, which depend on the wavelength and the chromophore’s absorption spectrum. The combination of using light illumination and US detection provides PAI with advantages (e.g., high sensitivity and specificity at a greater imaging depth) over other modalities that utilize only light or US for signal generation and detection.^39^^,^^59^ Optical imaging modalities, such as RCM and OCT, have shown the ability to differentiate tissue microstructures; however, they have limited penetration depth due to the scattering of light in tissue,^60^^–^^62^ even with performing enhancement postprocessing algorithms.^18^^,^^20^^,^^63^^–^^79^ US imaging provides improved penetration depth^80^ but has limited ability to differentiate between skin conditions.^81^

PAI can be classified by axial resolution of generated images into PA macroscopy, mesoscopy, and microscopy. Macroscopy utilizes lower US frequencies, which allows for greater penetration depths, in the range of a few centimeters.^82^^–^^84^ This has enabled noninvasive imaging of breast cancer,^85^ Crohn’s disease activity,^86^ brown fat metabolism,^87^ and various blood vessels located in the neck (carotid),^88^ hands,^89^ and feet,^90^ as well as whole-body imaging in animals.^91^^,^^92^ Microscopy utilizes higher US frequencies and allows for imaging with resolution capable of imaging individual cells^93^^–^^96^ but with much lower penetration depth. Mesoscopy is a bridge between macroscopy and microscopy, utilizing US frequencies that image at penetration depths of several millimeters,^82^ making it optimal for dermatological investigations.^97^ Mesoscopy has been used to visualize: skin morphology generally,^87^^,^^98^^,^^99^ vascular patterns, morphology in psoriasis and atopic dermatitis,^100^ nailbed microvasculature,^101^ and vasodilation induced by hyperthermia.^102^

**Fig. 1:**
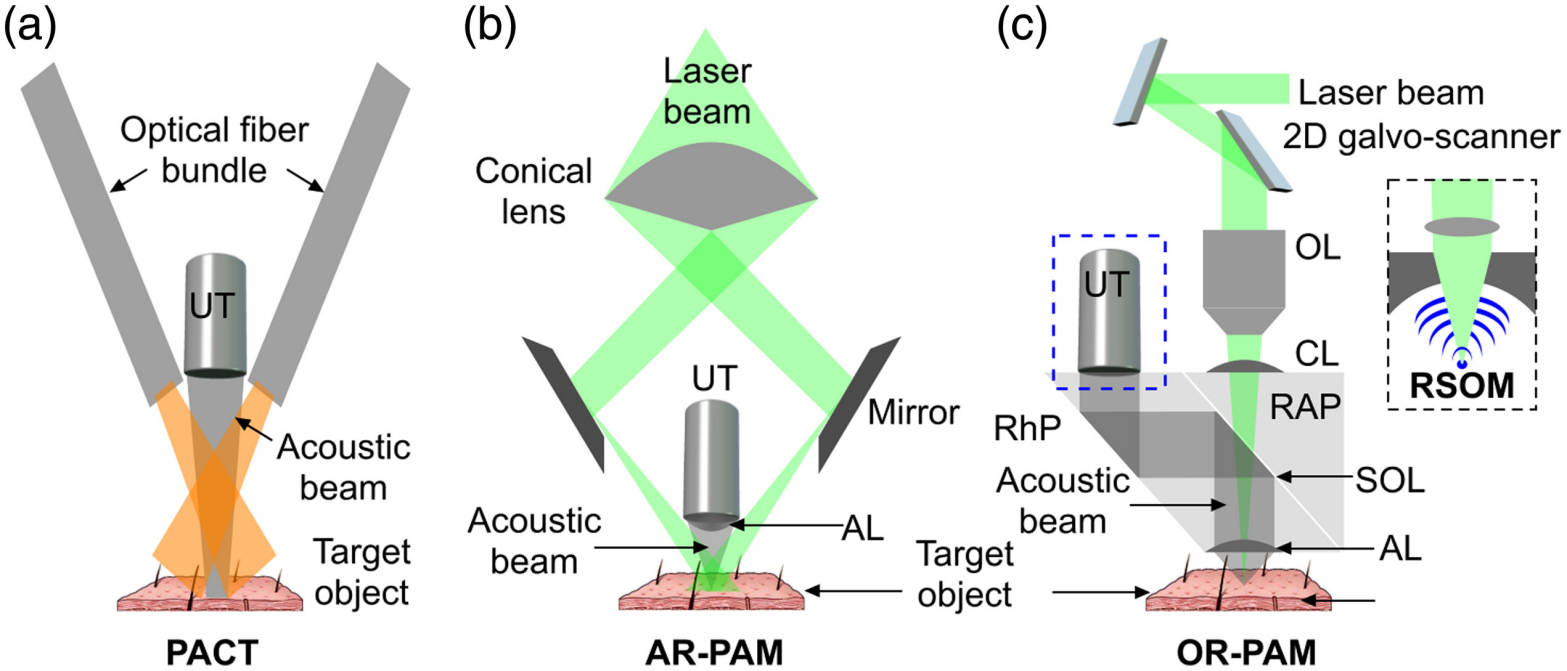
Different PAI configurations used for melanoma analysis: (a) PACT, (b) AR-PAM, and (c) OR-PAM (inset: RSOM). PACT, photoacoustic computed tomography; AR-PAM, acoustic-resolution photoacoustic microscopy; OR-PAM, optical-resolution photoacoustic microscopy; RSOM, raster scanning optoacoustic mesoscopy.

Another way to classify PAI systems is based on how the image is reconstructed (Fig. 1): in PA tomography (PAT), also known as PA computed tomography (PACT), a reconstruction algorithm is used to convert the PA signals collected at different locations/angles around the object into an image,^47^^,^^48^^,^^103^^,^^104^ whereas in raster scan PAI, the pixels/voxels of the image are generated as the sample is raster scanned. Based on the arrangement of transducers, PAT can further be categorized into single element PAT, linear array PAT (LA-PAT), ring array PAT, and hemispherical PAT,^47^^,^^48^^,^^103^^,^^105^^,^^106^ but LA-PAT is more widely implemented than other forms. Raster scan PAI has also been implemented in a variety of configurations,^47^^,^^107^^–^^109^ which can be divided into optical resolution PA microscopy (OR-PAM) and acoustic resolution PA microscopy (AR-PAM). In OR-PAM, the optical beam is focused to a tight spot, smaller than the acoustic detection sensitivity area and raster scanned point by point using an optical scanner.^110^^–^^112^ With OR-PAM, reaching to the cellular level resolution is achievable; however, the imaging depth is limited to a few millimeters.^108^ In AR-PAM, a spherically focused transducer scans the sample.^110^^,^^113^^,^^114^ This yields a greater penetration depth than OR-PAM but with a coarser resolution. Raster scanning optoacoustic mesoscopy (RSOM) is an implementation of AR-PAM that has also been used for skin imaging. Because raster scanning images samples point by point, image acquisition can be slow. Configurations for different PA systems (PACT, RSOM, OR-PAM, and AR-PAM) are shown in Fig. 1.

A third way to categorize PAI systems is whether they use a single wavelength laser to collect images or multiple wavelengths. In general, a single wavelength cannot distinguish between different chromophores.^92^ In multispectral PAI (MPAI), two or more wavelengths^92^^,^^115^^–^^119^ are used to differentiate between chromophores. For instance, melanin and hemoglobin can be differentiated one from another using 584 and 764 nm.^115^^,^^116^^,^^120^ MPAI has been used to study numerous diseases, including prostate cancer,^121^ glioblastoma,^122^^,^^123^ ovarian cancer,^124^ thyroid disease,^125^^,^^126^ Crohn’s disease,^86^ liver disease,^127^ cardiac injury,^128^ systemic sclerosis,^129^ and cutaneous tumors.^130^^,^^131^

**Fig. 2:**
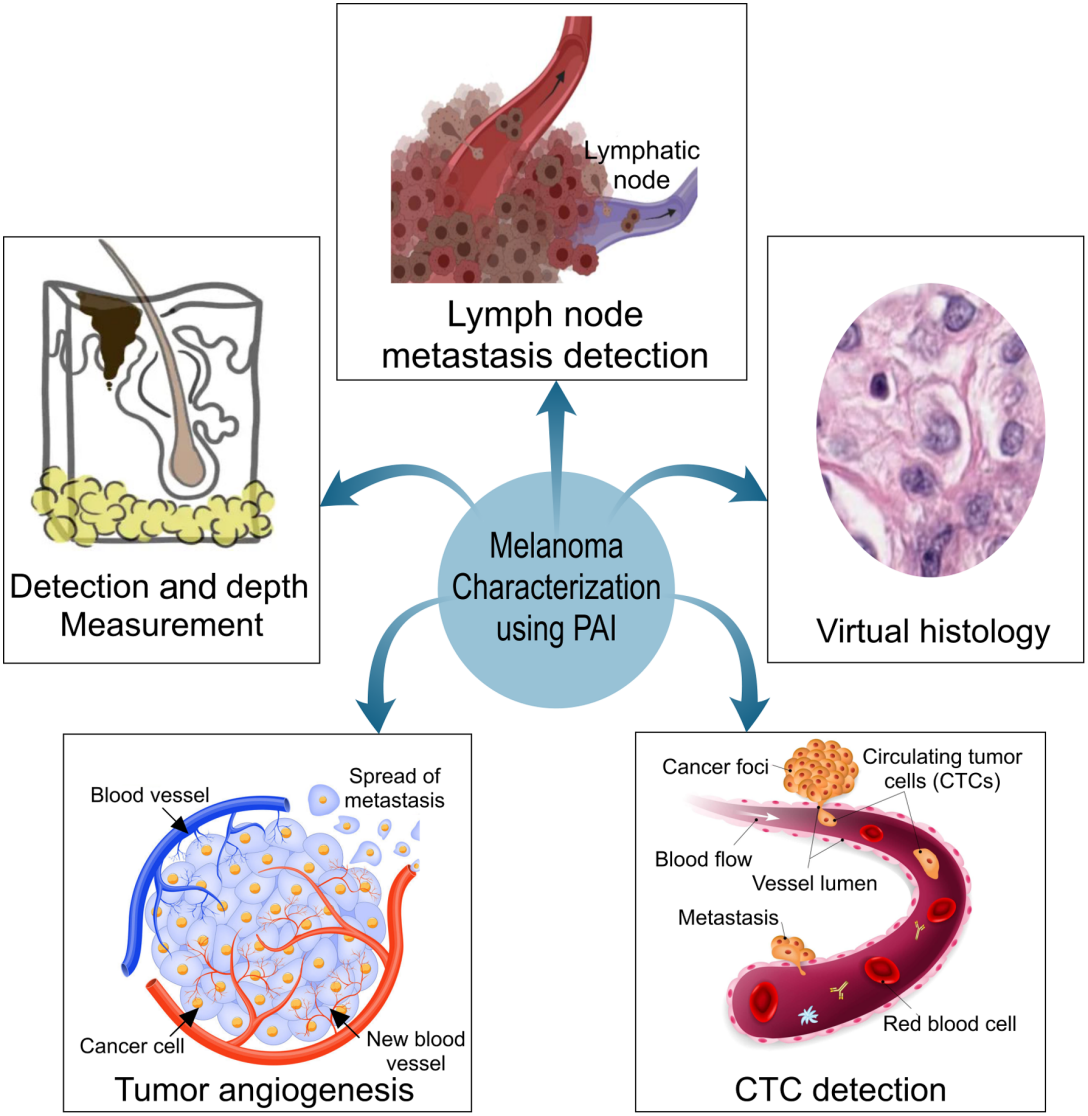
PAI applications for characterizing melanoma, discussed in this review. CTC, circulating tumor cells; PAI, photoacoustic imaging.

**Fig. 3:**
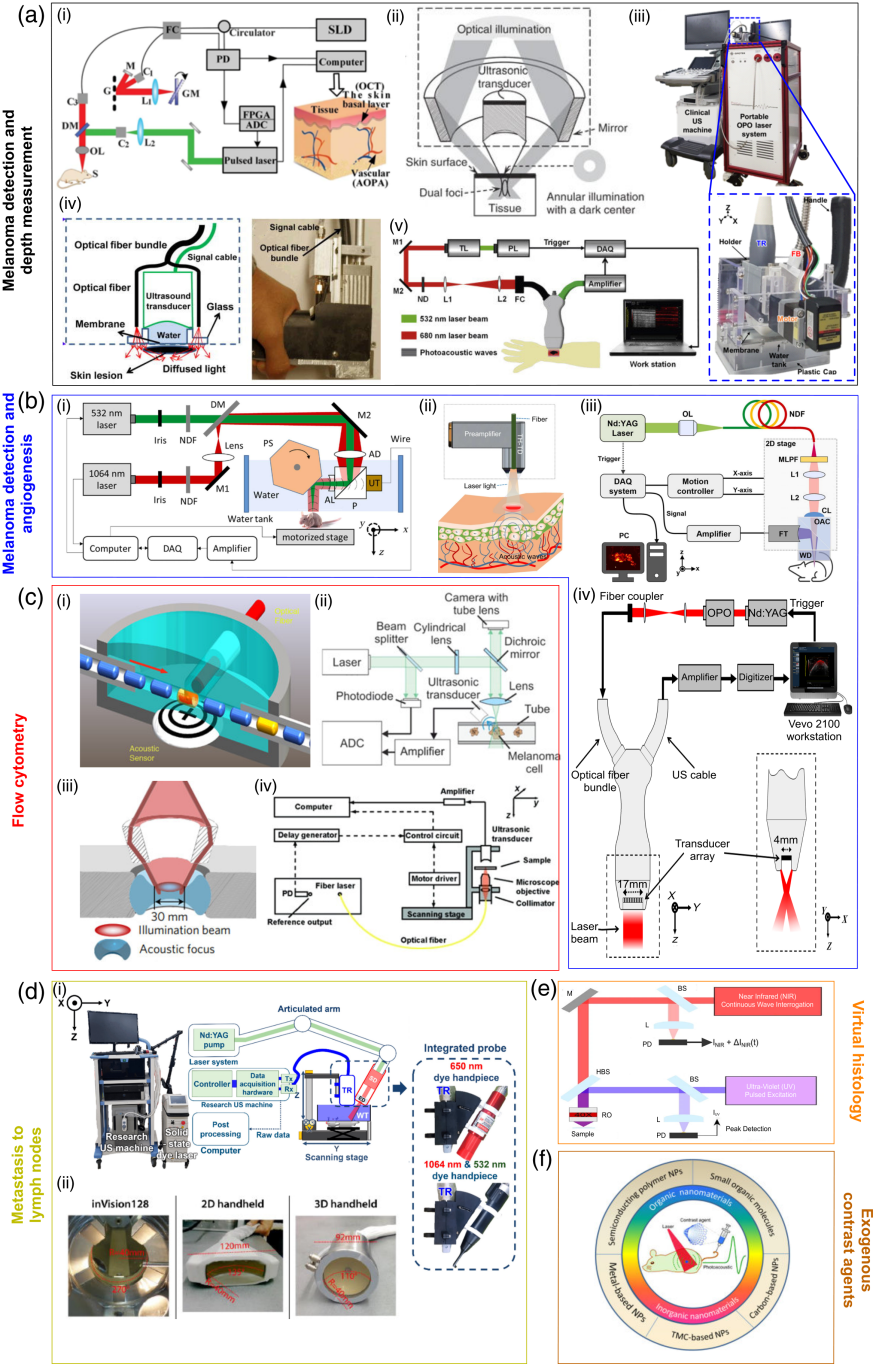
Hardware designs of selected PAI systems. (a) For melanoma detection and depth measurement: (i) all optical OR-PAM combined with OCT system. SLD, super luminescent diode; PD, photodetector; FC, fiber coupler; G, grating; GM, scanning galvanometer; $C_{1}$, $C_{2}$, and $C_{3}$, collimators; $L_{1}$ and $L_{2}$: lenses; M, mirror; DM, dichroic mirror; FPGA, field programmable gate array; ADC, analog-to-digital converter; OL, objective lens; S, sample; OCT, optical coherence tomography; AOPA, all-optical integrated photoacoustic. Reproduced from Ref. 132. (ii) Multiwavelength AR-PAM system. Reproduced from Ref. 113. (iii) 3D wide-field multispectral photoacoustic imaging of human melanomas, a dual-modal photoacoustic and US imaging system. Reproduced from Ref. 133. (iv) Multiscale PAM. NDF, neutral density filter; DM, dichroic mirror; $M_{1}$ and $M_{2}$, mirrors; AD, achromatic doublet; P, prism; AL, acoustic lens; UT, US transducer; PS, polygon-scanning mirror, DAQ, data acquisition. Reproduced from Ref. 134. (v) Handheld photoacoustic system for melanoma imaging. Reproduced from Ref. 135. (b) Melanoma detection and angiogenesis: (i) multiscale PAM. NDF, neutral density filter; DM, dichroic mirror; M_1_ and M_2_, mirrors; AD, achromatic doublet; P, prism; AL, acoustic lens; UT, US transducer; PS, polygon-scanning mirror, DAQ, data acquisition. Reproduced from Ref. 134. (ii) Schematic of faster raster scanning optoacoustic mesoscopy (RSOM). Reproduced from Ref. 109. (iii) Multiwavelength OR-PAM system. OL, objective lens; NDF, neutral dispersion fiber; MLPF, motorized long-pass filter; $L_{1}$ and $L_{2}$, achromatic lenses; CL, corrective lens; FT, focused transducer; WD, water dish; DAQ, data acquisition; OAC, optical/acoustic combiner. Reproduced from Ref. 136. (iv) Multispectral LA-PAT system. OPO, optical parameter oscillator. Reproduced from Ref. 115. (c) Flow cytometry: (i) single wavelength photoacoustic flow cytometry. Reproduced from Ref. 137. (ii) Multispectral photoacoustic flow cytometry. ADC, analog-to-digital converter. Reproduced from Ref. 138. (iii) Single-impulse panoramic photoacoustic computed tomography. Reproduced from Ref. 139. (iv) Optical resolution photoacoustic flow cytometry system. PD, photodiode. Reproduced from Ref. 140. (d) Metastasis to lymph nodes: (i) photograph and schematic of dual-modal photoacoustic and ultrasound imaging system with a solid-state dye laser. US, ultrasound; Tx, transmit; Rx, receive; TR, transducer; WT, water tank; ED, engineered diffuser; SD, solid-state dye. Reproduced from Ref. 141. (c) Multispectral optoacoustic tomography system with three different transducers used in the study, inVision 128, 2D handheld and 3D handheld. Reproduced from Ref. 117. (e) Virtual histology: system diagram of combined UV-PARS and UV scattering microscopy system. M, mirror; L, lens; BS, beamsplitter; HBS, harmonic beamsplitter; RO, reflective objective; PD, photodiode. Reproduced from Ref. 142. (f) Exogenous contrast agents: types of contrast agents utilized, implemented in animal studies. Reproduced from Refs. 143 and 144.

In terms of applications, PAI can also be categorized with regards to the type of questions that the imaging modality can be used to answer, namely, fitness of each PAI system to determine: (a) melanoma detection and depth measurement, (b) tumor angiogenesis, (c) lymph node metastases, (d) CTCs, and (e) virtual histology (see Fig. 2). Exogenous contrast agents have also been applied for some of these applications. Hardware designs of selected PAI systems that have been used in some of these applications are shown in Fig. 3. This categorization is consistent with the organization of the paper.

## 2 Methods

Several reviews have been written on skin imaging applications of PAI,^45^^,^^46^^,^^91^^,^^145^^–^^164^ but only one, that we are aware of, on melanoma imaging applications of PAI, and it is restricted to PAT applications and was published several years ago.^158^ We therefore conducted a literature review of primary research describing PAM and PAT to investigate CM that were published before December 2023. The literature search was conducted using the terms “PA” and “optoacoustic” with “melanoma.” Studies were excluded if they (1) did not utilize PA or optoacoustic methods, (2) were on cancers other than CM, (3) were not in English, (4) were not primary research papers, or (5) did not describe the parameters of the PA system utilized in the study. Our primary source was Google Scholar, which yielded 12,300 total results. Of the 12,300 results, 102 studies^103^^,^^109^^,^^113^^,^^115^^–^^117^^,^^132^^–^^140^^,^^165^^–^^245^ met our criteria and are reviewed here.

## 3 Investigations of CM Using PAI

### 3.1 Melanoma Detection and Depth Measurement

Imaging techniques able to detect melanoma with high sensitivity and specificity could greatly reduce the number of biopsies that are currently performed. Once a melanoma is confirmed through detection, the next step is melanoma staging. Staging melanoma is critical since it determines prognosis and treatment options.^12^ Melanoma depth (Breslow depth) is one of the two variables used to stage localized melanoma;^12^ the other one is ulceration status. The tumor (T) category of the tumor-node-metastasis staging system depends on tumor depth and is classified as T1 (≤1.00  mm), T2 (1.01 to 2.00 mm), T3 (2.01 to 4.00 mm), or T4 (>4.00  mm).^12^ For T1 melanomas, the 10-year survival rate is ∼92%, but drops to 50% for T4 melanomas.^12^ Excisional biopsy is required for staging lesions to accurately determine the Breslow depth.^11^^,^^246^^–^^250^ Imaging modalities could be used to stage melanoma, guide surgical planning, and prevent incomplete excisions and subsequent additional surgeries.

#### 3.1.1 Animal studies

**Fig. 4:**
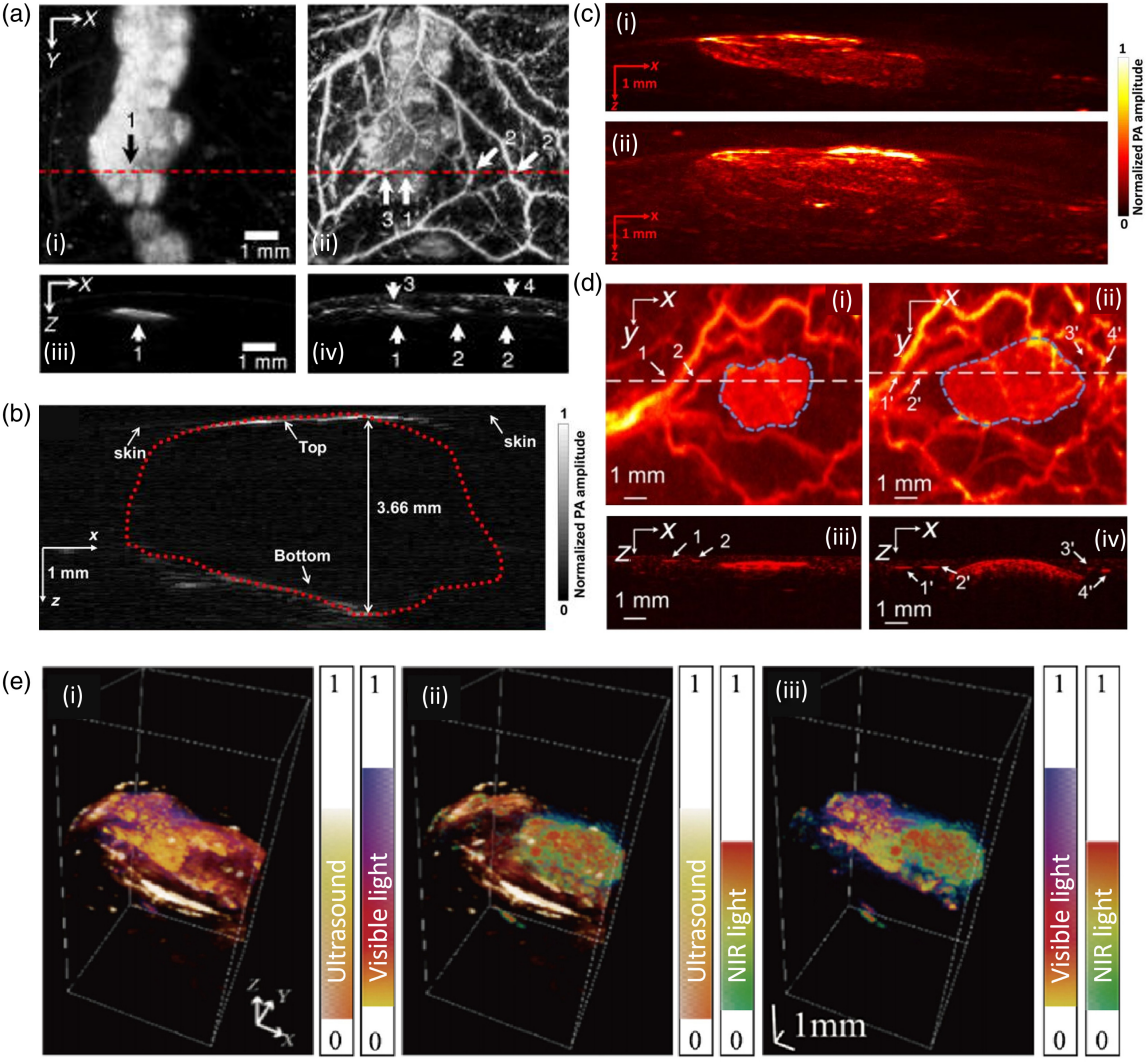
Melanoma detection and depth measurement in live animal models using different PAI configurations. (a) PA images of melanoma and vascular distribution in nude mouse skin. (i), (ii) En face PA images for the NIR light source (λ=764  nm) and visible light source (λ=584  nm) respectively: 1, melanoma; 2, vessels perpendicular to imaging plane; 3, vessels horizontal to imaging plane; 4, skin. (iii), (iv) B-Scan PA images along the red line in panels (i) and (ii). Reproduced from Ref. 116. (b) PA image of the melanoma showing both the top and bottom boundaries in nude mice *in vivo*. The red dots outline the melanoma. Reproduced from Ref. 188. (c) LA-PAT images of melanoma acquired in nude mice on day 3 (i) and day 6 (ii) after tumor implantation. Reproduced from Ref. 187. (d) PA maximum amplitude projection (MAP) images of melanoma in mice at (i) day 7, (ii) day 30 and PA B-scan images on (iii) day 7 and (iv) day 30. Reproduced from Ref. 184. (e) Fused 3D visible light and NIR PA MAP images in mice with melanoma. (i) visible light and US, (ii) NIR light and US, and (iii) visible and NIR light. Reproduced from Ref. 251.

Oh et al.^116^ used dual-wavelength AR-PAM to detect melanoma in mice inoculated with highly invasive B16 skin melanoma cells. Due to the difference in peak optical absorption between melanin and hemoglobin, a near-infrared (NIR) light (764 nm) and a visible light (584 nm) were used to visualize the melanoma [see Fig. 4(a)(i)] and surrounding vascular structures [see Fig. 4(a)(ii)]. Figures 4(a)(iii) and 4(a)(iv) illustrate the B-scan images across the red dotted line in Figs. 4(a)(i) and 4(a)(ii). The maximum thickness of the melanoma was found to be 0.5 mm. Zhou et al.^187^^,^^188^ conducted several experiments to study PAI in nude mice using a similar melanoma model. They used a handheld AR-PAM to measure the depth of melanoma.^188^ Their PAM utilized an “annular-shaped” light illumination method, in which light (with 8 mm inner diameter and 20 mm outer diameter) bypasses the center of the tumor and instead is delivered in the direction normal to the surface.^188^ The melanoma depth (3.66 mm) as measured by their system [see Fig. 4(b)] corresponded well to the actual, postexcisional thickness (3.75 mm).^188^ In another experiment utilizing a LA-PAT system, tumor depth and volume were measured and revealed an increase in tumor depth and volume from day 3 [Fig. 4(c)(i)] to day 6 [Fig. 4(c)(ii)] after injection with the B16 cells.^187^ The depth increased from 1.32 to 2.77 mm and volume increased from 22.365 to $71.931\;{\mathrm{mm}}^{3}$. Moreover, the system was able to measure the rate of growth for both depth and volume of tumor. Recently, Wang et al.^184^ built a hybrid PA/US system with a sound-light coaxial/confocal design by punching a 2 mm diameter hole in the center of the transducer to deliver the laser light. Melanomas in mice were imaged *in vivo* at day 7 [see Fig. 4(d)Ii)] and day 30 [see Fig. 4(d)(ii)] after B16 cell injection. A clear growth in tumor size and depth is observed as shown in B-scan images of melanoma at day 7 [see Fig. 4(d)(iii)] and day 30 [see Fig. 4(d)(iv)]. Moreover, PAI visualized microvasculature around the tumor. Another experiment with mice was conducted by Wang et al.^251^ using a dual-wavelength AR-PAM with visible and NIR light combined with US to image sub-CM. The fused images from the two wavelengths enrich the imaging information and allow more accurate detection of the melanoma, differentiating it from normal tissue. With the 3D distribution the boundary detection of the melanoma is easier and accurate and could be further enhanced by the US structural information helping the identification of the tissue boundaries and precisely locating the sub-CM [see Figs. 4(e)(i)–4(e)(iii)].

#### 3.1.2 Human studies

**Fig. 5:**
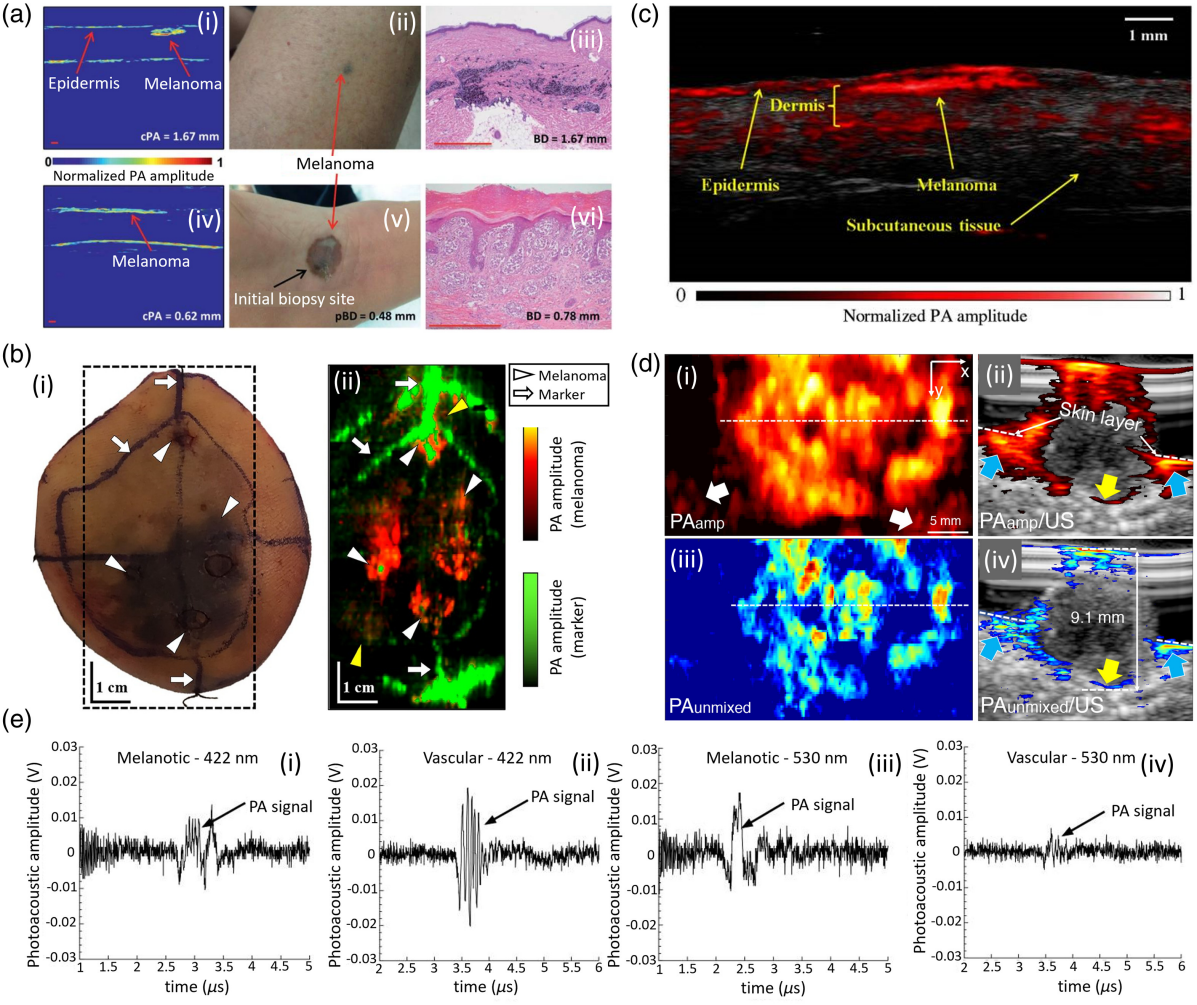
Melanoma detection and depth measurement in human studies using different PAI configurations. (a) PAT of melanoma of two patients: (i) melanoma image acquired with a PA depth of 1.9 mm (cPA depth=1.67  mm); (ii) cutaneous melanoma metastasis in a patient lower leg; (iii) histology of the excised melanoma, showing actual Breslow depth of 1.67 mm; (iv) PAT melanoma image of the acral lentiginous melanoma with PA depth of 0.70 mm (cPA depth=0.62  mm); (v) acral lentiginous melanoma (data not shown: pBD=0.48  mm); and (vi) histology after complete excision, with actual BD of 0.78 mm. BD, Breslow depth; cPA, corrected photoacoustic depth; PAT, photoacoustic tomography; pBD, provisional Breslow depth. Reproduced from Ref. 135. (b) (i) Photograph and (ii) *ex vivo* PA image of excised melanoma tissue from a male patient. The melanoma regions are represented by dark red to bright-yellow color (white triangles), and the marking-pen regions are represented by dark green to bright-green color (white arrows). Yellow arrows indicate possible melanomas not found by histology. Reproduced with permission from Ref. 177. (c) PA image of *in situ* melanoma on upper left extremity on a patient using LA-PAT. Reproduced with permission from Ref. 115. (d) Measurement of PA depth of a nodular type of melanoma. (i) Photoacoustic MAP; (ii) photoacoustic MAP, overlaid with US image; and (iii)–(iv) photoacoustic unmixed and photoacoustic unmixed overlaid with US images, respectively. Blue arrows indicate the melanoma invasion and yellow arrows are the bottom boundary of the melanoma. MAP, maximum amplitude projection. Reproduced from Ref. 133. (e) Representative PA signals from human subjects with either (i), (iii) pigmented and (ii), (iv) vascular lesions at 422 and 530 nm, respectively. Reproduced with permission from Ref. 252.

In a pilot study, Zhou et al.^135^ analyzed 10 melanomas in 7 patients using a LA-PAT system [see Fig. 5(a)] immediately preceding excisional biopsy. Melanomas at depths from 0.2 to 6 mm were visualized, but deeper tumors were beyond the detection limit of the LA-PAT system. Figure 5(a) shows the resultant PA images [see Figs. 5(a)(i) and 5(a)(iv)], photographs [see Fig. 5(a)(ii)–5(a)(v)], and histological images [see Figs. 5(a)(iii) and 5(a)(vi)] of two patients, one with CM metastases on the left lower extremity and the other with primary acral lentiginous melanoma on the right foot. Histological images acquired from both patients showed that the detected melanoma depth was consistent with actual Breslow depth, showing the promising capability of the system for detection. Kim et al.^177^ developed an integrated PA and US imaging (PAUSI) system by combining a clinical US machine and a multispectral portable laser. The imaging system was utilized to image a patient’s melanoma *ex vivo* after excision [see Fig. 5(b)(i)]. The amplitude of the PA signal corresponds to the amount of melanocytes in the local area. The PA signals in melanoma, indicated by the white triangles [see Fig. 5(b)(ii)], were predominantly generated by optical wavelengths of 800 and 1064 nm. In contrast, the PA signals from the marking pen regions indicated by the white arrows were predominantly generated from the 680-nm laser. Moreover, PA imaging indicated areas of melanoma that are not visible to the surgeon [yellow triangles in Fig. 5(b)(ii)]. In addition, the measured thickness of the melanoma region (420±320  μm) matched well with the histopathological results. A larger study on PAI was conducted by Breathnach et al.^115^ using LA-PAT and spectral unmixing, to pre-operatively image 32 pigmented lesions suspicious for melanoma in 27 patients. With spectral unmixing, they separated the absorption signature of melanin-containing cells and mapped the spatial distribution of it based on this signature, because the absorption spectrum of melanin varies within the NIR region. The lesion depths measured by PA were highly correlated with histopathologic measurements, with a correlation coefficient of 0.98 for benign lesions and 0.99 for melanomas. Using the same PA probe, the lesion architecture, adnexal depth (depth of lesion extension into the skin appendages), and various skin layers were also imaged, allowing for differentiation of superficial from invasive lesions based on their dermal-epidermal junction penetration, i.e., lesion penetrated through the dermal-epidermal junction [see Fig. 5(c)]. According to the authors, due to the tissue sample dehydration and loss of skin tension *in vivo*, PAI overestimated lesion depth as compared with histopathology. Recently, Park et al.^133^ utilized a 3D multispectral PAT system to noninvasively measure depth and outline the boundary of melanomas for optimal surgical margin selection. Six melanoma patients were examined. They imaged melanoma of various forms, sizes (1.3 to 30 mm for lateral diameter and 0.6 to 9.1 mm for depth), and locations (sole, chest, thigh, heel, and palm) using their multispectral PA/US system [see Fig. 5(d)]. For five of the six case studies, melanoma depth was measured using multispectral analysis and confirmed a high correlation against histopathologic results with a mean absolute error of 0.36 mm. In a signal-based study, Swearingen et al.^252^ investigated if label-free MPAI could distinguish vascular from pigmented (melanotic) lesions in 15 human patients. Excitation lights at 422 and 530 nm were used. At 422 nm, melanotic lesion showed a lower PA signal [see Fig. 5(e)(i)] compared with vascular lesion [see Fig. 5(e)(ii)]. Similarly, at 532 nm, melanotic lesion showed higher PA signal [see Fig. 5(e)(iii)] compared with vascular lesion [see Fig. 4(e)(iv)]. The experiment proved the ability of MPAI to distinguish between vascular and pigmented lesions. About 15 lesions were biopsied after imaging, revealing 8 vascular and 7 pigmented lesions. Data analysis was carried out via two statistical methods, the classical method (standard multivariate analysis classification techniques) and a Bayesian-model-based approach. The classical method attained a perfect lesion diagnosis rate, whereas the Bayesian approach had a 20% error rate.

Figures 4 and 5 and the related text describe results from a selection of PAI studies of melanoma detection and depth measurement. Table 1 includes specific characteristics of the PAI systems used in published studies on melanoma detection/depth measurement for both animal and human studies.

**Table 1:** Specific characteristics of PAI systems used in published studies on melanoma detection/depth measurement.

PAI modality	Wavelengths	US transducer	Resolution	Imaging model	Study result
AR-PAM	λ1: 764 nm	CF: 50 MHz	LR: 45 μm	Mice (B16 subcutaneous injection)^116^	Dual wavelength melanoma imaging and depth calculation
	λ2: 584 nm	BW: 70%	AR: 15 μm		
					Melanoma depth:
		NE: 1			λ1: 2D: 0.3 mm and 3D: 0.5 mm
					λ2: 2D: 0.45 mm and 3D: 0.15 mm
AR-PAM	λ: 650 nm	CF: 25 MHz	LR: 230 μm	Mice (B16 subcutaneous injection)^188^	Annular-shaped light illumination method for melanoma detection
		BW: 100%	AR: 59 μm		
		NE: 1			
					Melanoma depth: 3.66 mm
AR-PAM	λ1: 422 nm	CF: 25 MHz	NL	Human subjects with melanoma^252^	Study and classification of 15 human subjects with eight vascular and seven pigmented lesions
	λ2: 530 nm	BW: 100%			
		NE: 1			
AR-PAM	λ1: 532 nm	CF: 25 MHz	LR: 0.17 mm	Mice (B16 subcutaneous injection)^251^	Detection of melanoma with a combination of multi-wavelength PA images and US
	λ2: 1064 nm	BW: 80%	AR: 0.12 mm		
		NE: 1			
OR-PAM	λ: 532 nm	CF: 29 MHz	LR: 3.5 μm	Mice (B16 subcutaneous injection)^184^	Characterization of tumor vasculature and depth
		BW: 80%	AR: 60 μm		
		NE: 1			Melanoma depth: 2.58 mm
OR-PAM	λ: 584 nm	CF: 100 MHz	LR: 5 μm	Mice (B16 implanted in ear)^111^	Differentiation of blood vessels and melanoma without contrast agent after 3 and 7 days of implantation
			AR: 15 μm		
		BW: NL			
		NE: 1			
OR-PAM	λ: 532 nm	CF: 125 MHz	LR: 0.8 μm	Melanoma cells fixed in formalin^210^	Detection and imaging of melanoma cells with high resolution *ex vivo*
		BW: 80%	AR: 7.6 μm		
		NE: 1			
OR-PAM	λ: 1064 nm	CF: 41 MHz	LR: 36 μm	Mice (B16 subcutaneous injection)^220^	Detection of melanoma without exogenous contrast agent
		BW=NL	AR: 61 μm		
OR-PAM	λ: 532 nm	CF: 13 MHz	LR: 8.5 μm	Mice (B16 implanted in ear)^231^	Detection of the boundaries of melanoma and depth profiling
		BW: 60%	AR: 150 μm		
		NE: 1			
OR-PAM	λ: 600 to 1000 nm	CF: 25 MHz	NL	Mice (B16 subcutaneous injection to torso and ear)^136^	Differentiating melanomas from blood vessels and monitoring melanoma growth
		BW: NL			
		NE: 1			
All optical OR-PAM	λ: 532 nm	Michelson detector	LR: 13 μm	Mice (B16 implanted in ear)^132^^,^^232^	Detection and monitoring of melanoma and angiogenesis with dual modality (PAM/OCT)
			AR: 20 μm		
SW-PAM	λ: 532 nm	CF: 40 MHz	LR: 400 nm	Mice (B16 implanted in ear)^205^	Detection of melanoma and visualization of nearby vasculature. Monitoring melanoma growth over 4 days
		NA: 0.5			
		NE: 1			
LA-PAT	λ: 680 nm	CF: 21 MHz	LR: 119 μm	Mice (B16 subcutaneous injection)^187^	Detection of melanoma depth and volume
		BW: 55%	AR: 86 μm		
		NE: 256			
LA-PAT	λ: 700 nm	CF: 45 MHz	LR: 8 μm	Mice (B16 subcutaneous injection)^237^	Monitoring melanoma volume after photodynamic treatment
		BW: 55%	AR: 15 μm		
		NE: 256			Melanoma depth: 4 mm
LA-PAT	λ: 680 nm	CF: 21 MHz	LR: 119 μm	Human subjects with melanoma^135^	Detection of melanomas with depths of 0.2 to 6 mm
	F: $10\;{\mathrm{mJ}/\mathrm{cm}}^{2}$	BW: 70%	AR: 86 μm		
		NE: 256			The detection limit was 10 mm
LA-PAT	λ1: 800 nm	CF: NL	LR: 1200 μm	Human subjects with melanoma^177^	Detection of melanoma and measurement of thickness of the melanoma region
	λ2: 1064 nm	BW: NL	AR: 205 μm		
	λ3: 680 nm	NE: 128			
					Melanoma depth: 420±320 μm
LA-PAT	λ1: 680 nm	CF: 40 MHz	NL	Human subjects with melanoma^115^	Testing feasibility of LA-PAT probe and measuring tumor depth. High correlation with histopathology (0.99 for melanomas, 0.98 for benign lesions)
	λ2: 700 nm	BW: 55%			
	λ3: 750 nm	NE: 256			
	λ4: 850 nm				
	λ5: 900 nm				
LA-PAT	λ: 700 nm	CF: 40 MHz	LR: 140 μm	Human subjects with melanoma^218^	Detection of melanoma and identification and measurement of lesion boundaries
	λ: 850 nm	NE: 256			
LA-PAT	λ: 680 to 970 nm	CF1: 20 MHz	LR1: 110 μm	Human subjects with melanoma (*ex vivo* samples)^103^	Feasibility of PAI for noninvasive delineation of the borders of melanoma
		BW1: 55%	AR1: 50 μm		
		CF2: 30 MHz	LR2: 165 μm		
		BW2: 80%			
			AR2: 75 μm		
PAT	λ1=700 nm	CF: 7.5 MHz	LR: 1.0 mm	Human subjects with melanoma^133^	Measurement of melanoma depth and confirmation of metastastatic melanoma
	λ2=756 nm	BW: 120%	AR: 0.2 mm		
	λ3=796 nm				
	λ4=866 nm	NE: 128			Melanoma depth: 9.1 mm
	λ5=900 nm				

Note: λ, wavelength; F, fluence; CF, central frequency; BW, bandwidth; NE, number of elements; NL, not listed; IA, incident angle; LR, lateral resolution; AR, axial resolution; LA-PAT, linear array photoacoustic tomography; AR-PAM, acoustic resolution photoacoustic microscopy; OR-PAM, optical resolution photoacoustic microscopy; SW-PAM, subwavelength resolution photoacoustic microscopy.

### 3.2 Measurement of Tumor Angiogenesis in Melanoma

**Fig. 6:**
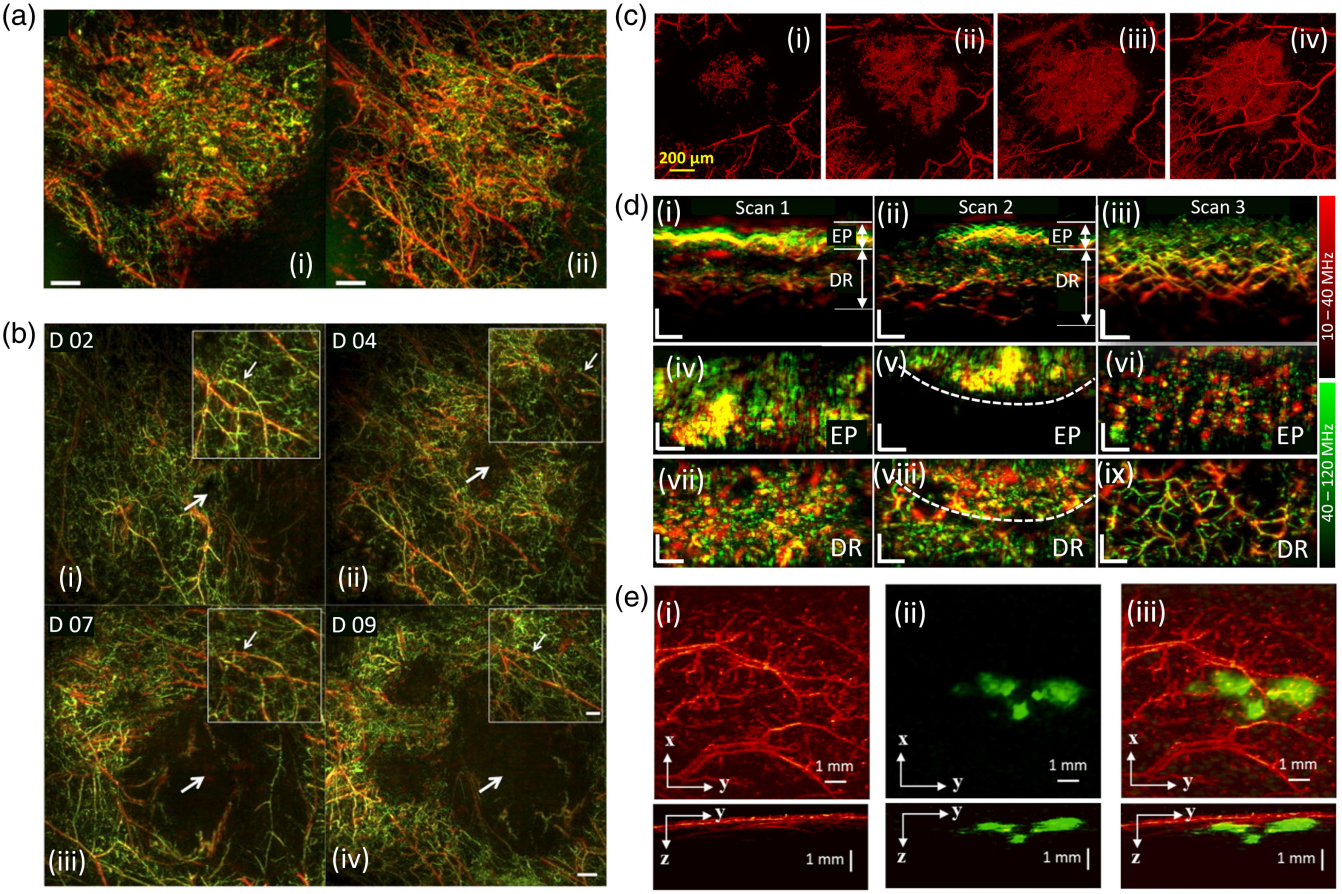
Angiogenesis studies in melanoma identification. (a) PA imaging of melanoma tumor vasculature in mice using (i) 50 MHz and (ii) 100 MHz detector. Reproduced with permission from Ref. 182. (b) PA images showing tumor growth over time. PA images of the tumor area acquired at (i) day 2 (D02), (ii) day 4 (D04), (iii) day 7 (D07), and (iv) day 9 (D09) after injection of melanoma cells, the thick arrow points to the tumor. The inset in every image shows the same region, from an area very close to the tumor, and shows two large vessels (the inset arrow points to that region), between which small vessels grow over time. Reproduced with permission from Ref. 182. (c) PA vascular imaging of melanoma in mouse at (i) day 9, (ii) day 13, (iii) day 14, and (iv) day 15. Reproduced with permission from Ref. 185. (d) PA images of human melanoma *in vivo* for three scanned regions: scan 1: inside the lesion, scan 2: boundary of the lesion, and scan 3: outside the lesion. (i)–(iii) Maximum amplitude projection (MAP) cross-sectional faster raster-scan optoacoustic microscopic (FRSOM) images. (iv)–(vi) MAP images in the coronal direction corresponding to the epidermal layer in panels (i)–(iii). (vii)–(ix) Coronal images corresponding to the dermal layer. EP, epidermal; DR, dermal. Reproduced with permission from Ref. 109. (e) *In vivo* OR and AR images of melanoma in mouse using multiscale PAM. (i) Top and side view MAP images obtained by OR-PAM at 532 nm. (ii) Top and side view MAP images obtained by AR-PAM at 1064 nm. (iii) Merged images of top and side view from OR and AR-PAM. Reproduced with permission from Ref. 134. OR, optical resolution; AR, acoustic resolution.

Tumor angiogenesis refers to the formation of new blood vessels within a tumor, or the growth of blood vessels between a tumor and its surrounding tissues. Tumor-associated vasculature not only regulates the supply of nutrients and oxygen to the tumor but also expedites tumor invasion and metastasis. Therefore, as an essential indicator of disease progression, vascularization within a tumor can be used to assess the potential for metastasis.^184^^,^^253^ PAI has shown great promise in monitoring the progression of tumor angiogenesis^143^^,^^254^ using OR-PAM and also RSOM in mice and humans (Table 2 and Fig. 6).

**Table 2:** Summary of PAI studies on melanoma tumor angiogenesis.

PAI modality	Light source	US transducer	Resolution	Imaging model	Study result
RSOM	λ: 532 nm	CF1: 50 MHz	LR: 18 μm	Mice (B16 subcutaneous injection to mammary pad)^182^	Studying changes in the vascular network caused by melanoma
		BW1: 160%	AR: 4 μm		
		CF2: 100 MHz			
		BW2: 160%			50 MHz: superior imaging of larger structures
					100 MHz: better visualization of tumor microvasculature
All optical PAM/RCM	λ: 532 nm	Low-coherence interferometer:	LR: 4.5 μm	Mice (B16 implanted in ear)^186^	Dual modality system
					PAM: visualized vascularity and pigmentation
		CW: 1310 nm			
		SB: 45 nm			RCM: illustrated cytological features
OR-PAM	λ1: 570 nm	CF: 75 MHz	NL	Mice (B16 implanted in ear)^185^	Detection of changes in tumor vascularity from 9 to 15 days after tumor inoculation
	λ2: 1064 nm	BW: NL			
OR-PAM	λ: 610 nm	PVDF	LR: 6 μm	Mice (B16 implanted in ear)^234^	Detection of neovasculature surrounding the tumor 9 days after melanoma inoculation
		CF: 25 MHz	AR: 53 μm		
OR-PAM	λ1: 730 nm	CF1: 7.5 MHz	LR: 102 μm	Mice (B16 subcutaneous injection)^241^	Quantification of the angiogenesis and melanoma thickness
	λ2: 756 nm	CF2: 31.5 MHz	AR: 81 μm		
	λ3: 778 nm				
	λ4: 796 nm				
	λ5: 818 nm				
OR-PAM	λ1: 528 nm	CF: 50 MHz	LR: 7.8 μm	Mice (B16 implanted in ear)^233^	Identification of the vasculature around the tumor with high resolution
	λ2: 558 nm	BW: 76%	AR: 41 μm		
		NE: 1			
OR-PAM	λ1: 532 nm	CF: 50 MHz	LR OR: 7.1 μm	Mice (B16 subcutaneous injection to thigh)^134^	Rapid visualization of melanoma boundaries. Imaging of the blood vasculature around the melanoma
	λ2: 1064 nm	BW: 70%	LR AR: 112 μm		
		NE: 1	AR: 10 μm		
AR-PAM	λ1: 584 nm	CF: 50 MHz	LR: 45 μm	Mice (B16 subcutaneous injection)^113^	Identification of melanoma and angiogenesis surrounding the tumor
	λ2: 764 nm	BW: 70%	AR: 15 μm		
RSOM	λ: 532 nm	CF: 25 MHz	NL	Humans with melanoma^109^	Quantifiable biomarkers were extracted from the vascular images by comparing benign nevi and melanomas
		BW: 120%			
		CF: 80 MHz			
		BW: 100%			
PARS	λ: 532 nm	Low coherence interrogation laser	LR: 2.7 μm	Chicken eggs with melanoma^227^	Study of superficial microvasculature with high lateral resolution
		CW: 1310 nm			
		CL: 40 μm			

Note: λ, wavelength; F, fluence; CF, central frequency; CW, central wavelength; SB, spectral bandwidth; CL, coherence length; NL, not listed; RSOM, raster scanning optoacoustic mesoscopy; OR-PAM, optical resolution photoacoustic microscopy; RCM, reflectance confocal microscopy; PARS, photoacoustic remote sensing microscopy.

#### 3.2.1 Animal studies

In one study, Omar et al.^182^ utilized RSOM to visualize angiogenesis and tumor growth in melanomas of mice *in vivo* over several days. B16F10 melanoma cells were injected subcutaneously into the mammary fat pad of Hsd: Athymic $\mathrm{Nude}\text{-}\mathrm{Foxn}1^{\mathrm{nu}}$ mice, and tumor growth and angiogenesis were monitored using spherically focused US detectors with central frequencies of 50 and 100 MHz. The 50 MHz detector was superior in imaging larger structures such as larger, oblique blood vessels [see Fig. 6(a)(i)], whereas the 100 MHz detector provided better visualization of tumor [appearing as a black hole in Fig. 6(a)(ii)] and newly sprouting vessels [see Fig. 6(a)(ii)]. Using the 50 MHz detector, tumor growth was recorded at day 2 [see Fig. 6(b)(i)], day 4 [see Fig. 6(b)(ii)], day 7 [see Fig. 6(b)(iii)], and day 9 [see Fig. 6(b)(iv)]. Each subfigure in Fig. 6(b) includes an inset taken from the proximity of the tumor. The growth of the tumor from day 2 to day 9 was illustrated by the growth of the black nonvascularized spot, indicated by a thick arrow in all the subfigures in Fig. 6(b). Moreover, in each inset, it is observed that upon interaction with the tumor, the two big vessels (denoted by a thinner arrow) start re-arranging; at the same time, smaller vessels start growing in that region, clearly representing tumor angiogenesis at a microvasculature level. Zhao et al.^185^ utilized OR-PAM at two wavelengths (570 and 1064 nm) to study melanoma tumor angiogenesis. B16 melanoma cells were injected subcutaneously into the mouse ear and tumors were imaged on day 9 [see Fig. 6(c)(i)], day 13 [see Fig. 6(c)(ii)], day 14 [see Fig. 6(c)(iii)], and day 15 [see Fig. 6(c)(iv)] after tumor inoculation in two mice. In the earlier day postinoculation, small diameter (<25 to 30  μm) vessels were most prevalent. On the following 2 days, the number of large diameter vessels (50 to 95  μm) increased while the proportion of small diameter vessels decreased. On day 15, vessels with diameter >100  μm were visualized. Moreover, the vessel density, vessel tortuosity, and fractional dimension (quantitative parameters they used to assess tumor growth) also showed an overall upward trend from day 9 to day 15. Thus, the authors concluded that with the growth of melanoma, the vascular networks become stronger and complex, which was consistent with visual results in Fig. 6(c). Another study by Zhou et al.^186^ used a combined all optical PA microscopy system and RCM system to study tumor growth and angiogenesis in mice who received subcutaneous injections of B16 melanoma cells in the ear. Their PAM showed irregular and linear vascular patterns, likely representing neovascularization of the dermis. The RCM enface image illustrated widespread pagetoid cells with cytologic atypia and nucleated cells within the dermal papilla. The PAM provided significant contrast and penetration depth, based on optical absorption properties, and visualized vascularity and pigmentation, whereas RCM illustrated cytological features. On the other hand, Xu et al.^134^ developed an integrated OR/AR-PAM system for multiscale imaging capability with high-speed wide-field imaging based on a polygon scanner. The polygon has six aluminum-coated surfaces that reflect the light and the acoustic beams: in this way six repeated cross-sectional scans can be obtained in one rotation of the motor, increasing image acquisition speed. With the two modalities and using two different wavelengths (532 and 1064 nm) a sub-CM is detected and separated from the surrounding microvasculature [see Fig. 6(e)].

#### 3.2.2 Human studies

Recently, angiogenesis of human melanomas has been imaged by He et al.^109^ using a single-breath-hold faster RSOM system to visualize the microvasculature of pigmented melanocytic lesion [see Fig. 6(d)]. The images from three different regions: scan 1 inside the lesion [see Figs. 6(d)(i), 6(d)(iv), and 6(d)(vii)], scan 2 in lesion boundary [see Figs. 6(d)(ii), 6(d)(v), and 6(d)(viii)], and scan 3 outside lesion [see Figs. 6d(iii), 6(d)(vi), and 6(d)(ix)], showed a difference on the epidermis and dermis vasculature patterns inside and outside the lesion. From the scans of 10 dysplastic nevi and 10 melanomas, a quantification of the vasculature features was performed to identify biomarkers for melanoma detection. A total of six biomarkers were calculated: the total blood volume, vessel density, average vessel length, tortuosity, fractal number, and lacunarity. It was found that those markers showed differences between malignant and benign lesions, supporting the possibility to use this system to improve melanoma diagnosis.

### 3.3 Lymph Node Metastases

Melanoma survival rates are high when the disease is caught early, but decrease significantly after nodal or distant metastasis. The sentinel lymph node (SLN) is the hypothetical first lymph node draining the cancer; SLN metastasis has been shown to predict the pathologic state of the nodal basin.^11^ An SLN biopsy is typically performed during wide local excision if the Breslow depth is >1  mm; moreover, SLN biopsies are considered in ulcerated tumors of any depth.^11^^,^^255^ The SLN for a specific melanoma is identified through pre-operative lymphoscintigraphy, or intra-operatively using either a blue dye injection near the primary tumor site or a gamma probe with technetium-99 sulfur colloid.^11^ After excision, SLNs are examined via histopathology and immunohistochemistry for metastasis. However, these examinations can miss the presence of metastases due to false negative rates varying from 5% to 21%.^256^ Once a lymph node is removed, typically 6 to 10 sections of ∼6  μm thickness are taken and examined for metastasis. Thus, in a typical node with 1 cm length, there are hundreds of possible sections, only a fraction of which are histologically examined.^176^ On the other hand, the presence of metastasis in an excised node can be determined by PAI^257^ since most melanomas are highly melanotic, containing ∼95% melanin,^11^ and melanin is a highly absorbing chromophore in NIR range.^115^ Therefore, SLN metastases can be accurately identified by PAI, leading to appropriate selection of sections for histological examination, mitigating the possibility of false negative results.^258^

#### 3.3.1 Animal studies

**Fig. 7:**
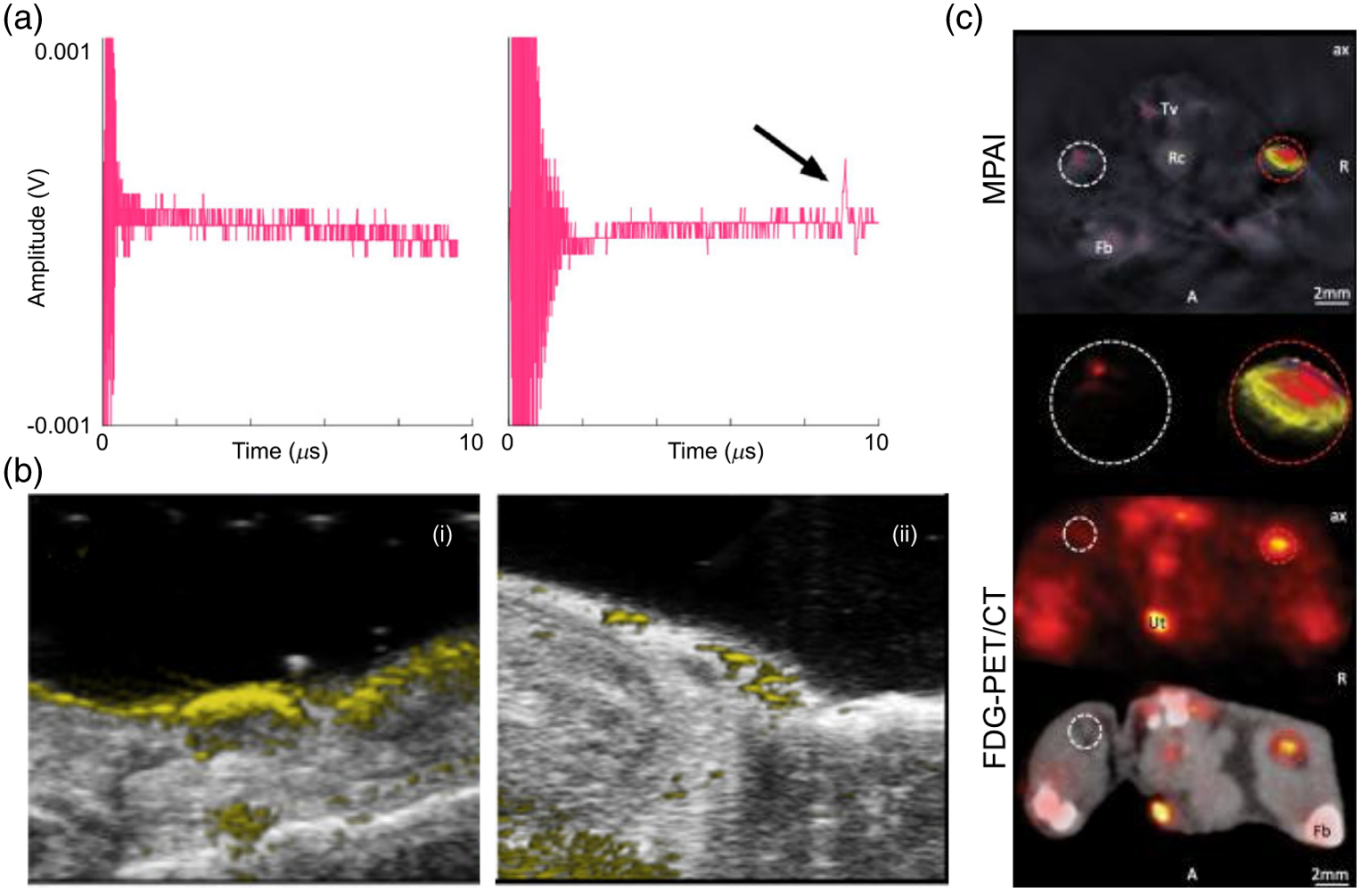
Animal studies of lymph node metastases analyzed by PAI. (a) Photoacoustic response from lymph nodes (i) without melanoma, the detector shows no signal with a noise floor of 100  μV, and (ii) with melanoma, showing a photoacoustic wave at 5  μs. Reproduced with permission from Ref. 176. (b) Comparative results between multispectral PAT and FDG-PET/CT systems for detection of macrometastasis in right popliteal lymph node in mice. The red circle encloses the melanoma macrometastasis region. White circle enclosed the contralateral popliteal healthy control node. Multispectral images were unmixed for deoxyhemoglobin (blue), oxyhemoglobin (red) and melanin (yellow). Fb, femur; Tv, tail vessel; Rc, rectum; Ut, urethra. Reproduced with permission from Ref. 180. (c) *In vivo* images of melanin-specific photoacoustic signal with grayscale underlay of a lymph node that (i) shows metastasis at 60 days post tumor induction and (ii) negative control mouse that did not undergo tumor induction. MPAI, multispectral photoacoustic imaging; FDG-PET/CT, fluorodexyglucose PET/CT. Reproduced with permission from Ref. 183.

PAI has been studied for the imaging of lymph nodes (Table 3) to identify metastases both *ex vivo* (postsurgical resection) and *in vivo*. McCormack et al.^176^ used a three-single-element-transducer based PAT prior to and after injecting a human melanoma cell line (HS 936) into *ex vivo* lymph nodes from a healthy canine and pig. The PAT system they used consisted of a 600  μm diameter optical fiber connected to a tunable laser and an acoustic sensor made from polyvinylidene fluoride film. The 532 nm wavelength light was illuminated from the top of the lymph node, and the PA signals were detected from the bottom [see Fig. 7(a)]. The control lymph nodes (with no injected melanoma cells) showed no PA response [see Fig. 7(a)(i)], whereas the melanoma cells in the excised lymph nodes generated PA signals as shown with the arrow in Fig. 7(a)(ii). A pig lymph node with only 500 injected melanoma cells and a 100 to 200  μm diameter lymph node produced a PA response. Neuschmelting et al.^180^ utilized a multispectral (wavelengths 700 to 860 sampled) PAT system with a cylindrically focused transducer array (iThera Medical) and compared it with fluorodexyglucose (FDG) positron emission tomography (PET)/CT for imaging B16F10-derived melanoma micrometastases and macrometastases to lymph nodes and in-transit metastases (metastases moving from the primary tumor to the nearby nodal basin) in mice *in vivo*. The PAT system detected lymph node micrometastases in the cortical region of lymph nodes (after 2 weeks’ tumor cell inoculation) that were too small for FDG PET/CT to detect. It also delineated in-transit metastases, which were observed as bright clusters between the primary melanoma site and the nodal basin. Both PAT and PET/CT could detect macrometastases [see Fig. 7(b)], but only the PAT system unmixed signals to enable detection of micrometastatic infiltration of melanoma in the cortex of popliteal nodes (white dashed circles). This multispectral PAT system distinguished melanoma lymph node metastases from other neoplastic and nonneoplastic lymphadenopathies (due to melanin’s contrast) but FDG PET/CT could not (due to nonspecific FDG uptake and relatively low resolution). Most recently, Sinnamon et al. utilized a Vevo LAZR PAT system to visualize inguinal lymph node metastases *in vivo* at 4 and 8 weeks after inoculation with B16 melanoma cells in the flank region in BRaf-PTEN transgenic mice.^174^^,^^183^ In total, 49 lymph nodes were imaged in 25 mice, with metastatic cells present in 17 lymph nodes (35%), with histopathological confirmation. Thus the system was able to image *in vivo* melanin PA signals of a lymph node containing melanoma metastasis at 60 days after the induction of the tumor [see Fig. 7(c)(i)]. The negative control mouse without tumor induction is shown in Fig. 7(c)(ii). The PA melanin signal within the positive lymph nodes was significantly higher than in the negative control mouse, with no difference in PA signals between the adjacent soft tissue of positive and negative lymph nodes. The strongest predictor of melanoma metastasis was the ratio of lymph node to soft tissue PA melanin peak signal.

**Table 3:** Summary of *ex vivo* and *in vivo* human and animal PAI studies for detection of melanoma metastasis to lymph nodes.

PAI modality	Light source	US transducer	Imaging model	Study result
LA-PAT	λ: 680 to 970 nm	CF: 21 MHz	Human lymph nodes imaged both *ex vivo* and *in vivo*^174^	Identification of melanoma metastasis in human lymph nodes
		BW: 70%		
		NE: 256		
LA-PAT/multispectral	λ: 700 to 860 nm	CF: 5 MHz	Mouse lymph nodes imaged *in vivo*^180^	Detection of lymph node micro metastases and in-transit metastases from melanoma
		BW: NL		
		NE: 256		
Vevo LAZR	λ: 680 to 970 nm	LZ250	Mouse lymph nodes imaged *in vivo*^183^	Melanin detection in melanoma metastases. The strongest predictor was the ratio of lymph node to soft tissue PA melanin peak signal
		CF: 21 MHz		
		BW: 55%		
		NE: 256		
		LZ201		
		CF: 15 MHz		
		BW: 60%		
		NE: 256		
PAT	λ: 532 nm	2.5 μm thick PVDF film and coaxial cable	Pig and canine lymph nodes imaged *ex vivo*^176^	Detection of melanoma micro metastases in SLNs
PAT	λ: 720 to 800 nm	CF: 6.25 MHz	Human lymph nodes imaged *ex vivo*^169^	First human study on melanoma lymph node metastasis. Examining the status of resected lymph nodes
		BW: >80%		
		NE: 32		
Photoacoustic finder	λ: 532 nm	CF: 8 MHz	Mouse lymph nodes imaged *in vivo*^239^	Use of PA for localization of the SLNs in rats without exogenous agent
	λ: 650 nm	CW: 45%		
		NE: 1		
PAT	*Ex vivo*:	CF: 5 MHz	Human lymph nodes imaged *ex vivo* from patients with stage I and II melanoma^117^	Imaging of SLNs to determine metastatic status
	λ: 700–880 nm	BW: 60%		100% sensitivity and 62% specificity
		NE: 128		
PAT	*In vivo*:	2D:	Human lymph nodes imaged *in vivo* from patients with stage I and II melanoma^117^	ICG dye used to mark SLNs. 100% sensitivity and 48.6% specificity
	λ: 700, 730, 760, 800, 850 nm	CF: 4 MHz		
		BW: 52%		
		NE: 256		
		3D:		
		CF: 2.5 MHz		
		BW: 60%		
		NE: 384		
LA-PAT	λ: 532, 650, 1064 nm	CF: 5.3 MHz	Rat lymph nodes imaged *in vivo*^141^	The SLN was detected in the rat beneath 2.2 cm thick chicken tissue layer, at a depth that is at least typical of SLN in humans
		BW: 90%		
		NE: 192		

Note: λ, wavelength; F, fluence; CF, central frequency; BW, bandwidth; NE, number of elements; NL, not listed; LA-PAT, linear array photoacoustic tomography; PAFC, photoacoustic flow cytometry; PAF, photoacoustic finder; AR-PAM, acoustic resolution photoacoustic microscopy; OR-PAM, optical resolution photoacoustic microscopy; SLN, sentinel lymph node.

#### 3.3.2 Human studies

**Fig. 8:**
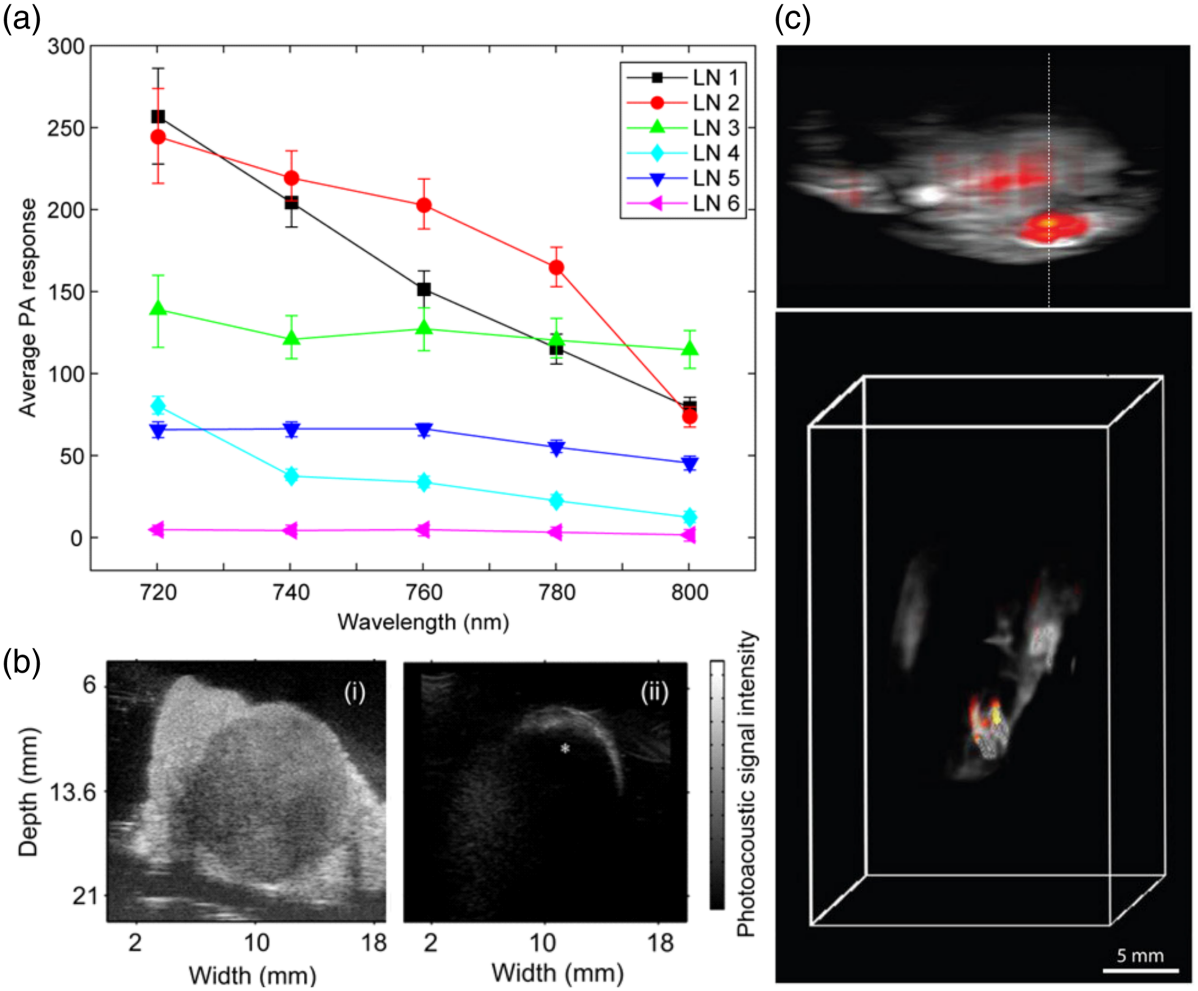
*Ex vivo* and *in vivo* studies of human lymph node metastases analyzed by PAI. (a) PA signal strength of the selected areas within the lymph nodes (LN) at different illumination wavelengths. Reproduced with permission from Ref. 169. (b) Images of two human nodes. (i) PA image of a metastatic node, (ii) PA image of a benign node. Absence of PA signal deeper in the malignant node [as indicated by * in panel (b)] seems to be caused by the strong absorption by the melanin in the superficial area of the node. Reproduced with permission from Ref. 174. (c) (i) Lateral MIP and (ii) 3D rendering image of *ex vivo* optoacoustic image of a human lymph node from a melanoma patient. Grayscale represents hemoglobin background and color bar overlay shows the multispectral resolved signals for melanin. Reproduced with permission from Ref. 117.

Many different PAI modalities have been utilized for *ex vivo* studies of metastases in melanoma SLNs, including LA-PAT and arc-shaped PAT.^117^^,^^169^^,^^174^ PAT has also been used for *in vivo* studies.^117^ The first human study on melanoma lymph node metastasis was conducted by Grootendorst et al.^169^ using a curvilinear array. Patients with proven metastatic disease undergoing inguinal or axillary lymphadenectomy were enrolled, and 1 or 2 lymph nodes were randomly selected for *ex vivo* multispectral analysis. The PA laser was illuminated from the top of the sample and the US detector array was rotated 360 deg around the sample. A total of six lymph nodes were imaged, and PAT revealed that three lymph nodes were metastatic and three were benign. All three malignant nodes (LN1, LN2, and LN3) displayed increased PA signals (from melanoma cells), whereas all three benign nodes had a substantially weaker signal (signal likely from hemoglobin and possibly other chromophores) when imaged at wavelengths from 720 to 800 nm [see Fig. 8(a)]. The results were confirmed through histopathology. Langhout et al.^174^ used a LA-PAT system and imaged 12 lymph nodes. Histopathology revealed that three nodes were metastatic and nine were benign. Again, melanoma-positive nodes [see Fig. 8(b)(i)] displayed different PA signals (depth image) than benign nodes [see Fig. 8(b)(ii)] due to the difference in melanin distribution. Additionally, total volume imaging to the depth of 2 cm in benign lymph nodes (absent melanin deposits) allowed for computation of the entire nodal volume. The largest human study on PAI of melanoma metastasis to lymph nodes was carried out by Stoffels et al.^117^ using a multispectral arc-shaped PAT. They analyzed 506 SLNs from 214 patients with a Breslow depth of at least 1 mm: 148 SLNs (from 65 patients) were analyzed by multispectral PAT *ex vivo* and histology, whereas the other 358 SLNs (from 149 patients) were analyzed by the conventional European Organization for Research and Treatment of Cancer (EORTC) melanoma group protocol. Their system detected metastases in 22.9% of excised SLNs compared with 14.2% by the EORTC Melanoma Group protocol. *Ex vivo* analysis by PAT showed 100% sensitivity and 62% specificity. Then, an *in vivo* experiment was carried out using PAT and indocyanine green (ICG) (a NIR fluorophore injected peri-tumorally) as a contrast agent to image 41 SLNs in 20 patients. PAT visualized ICG-marked SLNs to the depth of 5 cm (discerned by single photon emission computed tomography/CT) and with 100% concordance with the gold standard of SLN detection, 99 m Tc-nanocolloid–guided lymphoscintigraphy. *In vivo* PAT analysis revealed a sensitivity of 100% and specificity of 48.6%. With 100% sensitivity, both *ex vivo* and *in vivo* PAT identified noncancerous SLNs in 189 total lymph nodes without any false negatives but with a high rate of false positives. The quantification of melanin was performed with multispectral analysis and then correlated with the localization of metastatic cells [see Fig. 8(c)].

### 3.4 Metastases to the Blood: Imaging CTCs

CTCs originate in the primary tumor and acquire genetic and structural alterations, leading to changes in cellular signaling and protein expression. This results in bloodstream intravasation and possibly invasion to other organs to develop secondary tumors.^259^^,^^260^ Previous work has shown that CTC assays can predict prognosis and treatment response in patients with breast, prostate, and colorectal cancers.^261^^–^^263^ CTCs can be detected via flow cytometry, which has traditionally involved detecting fluorescent signals from cells labeled with multicolor probes *ex vivo*, which is known as conventional flow cytometry.^264^^,^^265^ However, this method requires invasive blood extraction from patients and is limited in its ability to detect infrequent CTCs.^265^ Additional CTC assays have been developed,^266^ but have not been studied on a large scale, limiting their clinical translatability.

Flow cytometry can be done *in vivo* and noninvasively using PA flow cytometry (PAFC). The concept of PAFC is similar to PAI: an excitation light is absorbed by chromophores in blood cells, causing local thermoelastic expansion and subsequent production of acoustic waves, which can be detected by an US transducer. PAFC configurations are an adaptation of OR-PAM, as high-pulse-repetition-rate lasers are also used in PAFC to generate detectable PA signals from individual CTCs.^265^ Higher pulse rates improve the signal-to-noise ratio (SNR) through averaging PA signals from individual CTCs; SNR is determined by the ratio of PA signals from individual CTCs to signals from other blood cells and background noise.^265^ The main chromophore in blood cells is hemoglobin in red blood cells (RBCs), but the main chromophore in melanoma CTCs is melanin. As with many other PAI modalities, in PAFC, multispectral analysis is often used to differentiate hemoglobin/melanin signals in the vasculature. Thus, unmixing of multispectral PA signals can allow for differentiation of CTC signals from oxy- and deoxy-hemoglobin.^167^ Numerous studies, summarized in Table 4, have investigated PAFC to detect melanoma CTCs.^137^^,^^139^^,^^166^^–^^168^^,^^170^^–^^172^^,^^178^^,^^179^^,^^189^^,^^190^

**Table 4:** Summary of *ex vivo* and *in vivo* human and animal PAI studies on melanoma CTCs.

PAI modality	Light source	US transducer	Resolution	Imaging model	Study result
PAFC	λ: 420 to 2300 nm	CF: 10 MHz	NL	*In vivo*: Imaging of the carotid artery of mice^168^	Develop *in vivo* lymph tests using the principles of flow cytometry. Calculated the CTC flow rate
		BW: NL			
		NE: 1			
PAFC	λ: 905 nm	CF: 3.5 MHz	LR: 100 μm	*In vivo*: Mice tail vein	Detection of CTCs. About 82% of unlabeled CTC were detected
		CF: 20 MHz	AR: 15 μm		
		BW: NL		Label: AuNR	
		NE: 1		Mice ears and skin. No label^167^	
PAFC	λ: 430 to 630 nm	PVDF transducer	NL	*In vitro* melan-oma cells tagged with AuNP^178^	The AuNP tagged melanoma cells showed 34% greater PA signal than the untagged cells
		BW: NL			
		NE: 1			
PAFC	λ: 820 to 1064 nm	CF: 3.5 MHz	NL	*In vivo*: Mice tail vein, ears vessels, and abdominal vessels^190^	Detection of CTCs in melanoma-bearing mice. PA signals were detected immediately after injection. Calculation of the CTC flow rate
		BW: NL			
		NE: 1			
PAFC	λ: 1060 nm	PVDF transducer	LR:	*In vitro*: Detection of CTCs spiked in blood samples. *In vivo*: Assessment of effect of optical clearing on mice and humans^179^	Detection of melanoma cells in blood flow
			40×1100 μm		
		CF: 16 MHz	AR: 20 μm		Optical clearing increased PA signal amplitude by 20% to 40%
		BW: 190%			
		NE: 1			
PAFC	λ: 532 nm	CF: 10 MHz	LR: 1.5 μm	*In vivo*: Mice, subcutaneous injection, detection of CTCs in ear^189^	Identification and count of RBCs and CTCs in the blood vessels
		BW: NL	AR: 132 μm		
		NE: 1			
PAFC	λ1: 532 nm	CF: 50 MHz	LR: 3 to 7 μm	*In vivo*: Mice, injection of B16 cells in jugular vein or carotid artery^172^	Detection of CTCs and clusters traveling in blood vessels
	λ2: 1064 nm	BW: NL	AR: 26 μm		
		NE: 1			
PAFC	λ: 1060 nm	PVDF	LR: 65 μm	*In vivo*: Healthy humans and humans with melanoma^166^	Detection of melanin-bearing CTCs in patients with melanoma. CTC flow rate was calculated. PAFC distinguished CTCs from circulating blood clots
		CF: 16 MHz			
		BW: 190%			
		NE: 1			
PAFC	λ1: 532 nm	PVDF transducer	NL	*In vitro*: Humans with melanoma^170^	Detection of CTCs. Signal from a single melanoma cell is detectable
	λ2: 680 nm				
		CF: NL			
		BW: NL			
		NE: 1			
PAFC	λ: 532 nm	PVDF transducer	NL	*In vitro*: Blood samples with cultured melanoma cells^207^	Detection of melanoma CTCs in blood samples
		CF: 50 MHz			
		BW: 100%			
		NE: 1			
PAFC	λ: 532 nm	NL	NL	*In vitro*: Blood samples of patients^137^	Detection of melanoma in stage IV patients
PAFC	λ: 1064 nm	CF: 20 MHz	NL	*In vitro and in vivo*: Mice blood or mice with injected B16 cells^213^	Detection of melanoma with nonlinear PAFC
		BW: NL			
		NE: 1			
PAFC	λ1: 532 nm	NL	LR: 30 μm	*In vivo*: CTCs in mice, rat tail artery^208^	PA detection of induced metastatic melanoma
	λ2: 622 nm				
PAFC	λ: 820 nm	CF: 10 MHz	NL	*In vivo*: Mice artery after tail injection^211^	Detection of strongly absorbing cells with positive contrast and negative contrast for fluorescence
		NE: 1			
PAFC	λ: 750 nm	CF: 40 MHz	LR: 40 μm	Gelatin phantom with solution containing CTCs^214^	Monitoring of low concentration of CTCs without labeling
		BW: 85%			
		NE: NL			
PAFC	λ1: 670 nm	CF1: 3.5	NL	*In vivo* and *ex vivo*: Mice brain^212^^,^^215^	Detection and counting of CTCs in CSF of tumor-bearing mice
	λ2: 820 nm	MHz			
		CF2: 20 MHz			
PAFC	λ: 1064 nm	CF: 3.5 MHz	NL	*In vitro* and *in vivo*: Melanoma cells in mouse blood and in mice paw^138^	Reliable detection of melanoma cells in the vessels after upstream arterial injection
		NE: 1			
PAFC	λ: 820 nm	CF: 2.25 MHz	NL	*In vivo*: Mice ears^193^	Detection of lymphatic CTCs in premetastatic disease
		NE: 1			
PAFC	λ: 532 nm	CF: 5 MHz	NL	*In vitro*: Human blood samples with stage I, II, and III melanomas^199^	Quantification of CTCs in serial blood samples at early stage melanoma to predict metastatic disease
		NE: 1			
OR-PAFC	λ: 1064 nm	CF: 40 MHz	LR: 15 μm	*In vitro*: Bovine blood samples with melanoma cells^140^	Detection of melanoma cells flowing in blood sample. Estimation of the flow speed of the cell in the fluid
		BW: 100%	AR: 37 μm		
		NE: 1			
LA-PAT	λ: 680 nm	CF: 21 MHz	LR: 119 μm	*In vivo*: Mice tail veins^171^	Detection and quantification of CTCs
		BW: 78%	AR: 86 μm		
		NE: 256			
LA-PAT	λ: 680 nm	CF: 40 MHz	LR: 94 μm	*In vivo*: Humans with melanoma^192^	Imaging suspected CTCs in patients *in vivo*, with a CNR >9
		BW: 85%	AR: 43 μm		
		NE: 256			
SIP-PAT	λ: 680 nm	CF: 5 MHz	SR: 125 μm	*In vivo*: Mice carotid artery^139^	Visualization of CTCs in mice cortical arteries and veins and calculation of the CTC flow rate
		BW: 90%			
		NE: 512			
PAT	λ: 700 nm	CF: 5 MHz	SP: 150 μm	*In vivo*: Mice brain with melanoma CTCs^191^	Detection and counting of individual CTCs in the CSF
		BW: 100%			
		NE: 512			

Note: RBC, red blood cell; λ, wavelength; CF, central frequency; BF, broadband frequency; BW, bandwidth; NE, number of elements; NL, not listed; AuNR, gold nanorods; LR, lateral resolution; AR, axial resolution; AR-PAFC, acoustic resolution PA flow cytometry; OR-PAFC, optical resolution PA flow cytometry; SIP-PAT, single pulse panoramic PAT; PVDF, polyvinyledene fluoride; CNR, contrast-to-noise ratio; CSF, cerebral spinal fluid.

#### 3.4.1 Animal studies

PAFC has demonstrated the ability to detect unlabeled (and labeled) CTCs in the mouse vasculature after injection of CTCs in the tail vein or from inoculation of primary melanoma tumors in the skin or ears.^87^^,^^167^^,^^168^^,^^172^^,^^189^^,^^190^ Overall, PAFC accurately detected CTCs from the background RBCs in the mouse vasculature, and PAFC signals increased as metastasis increased.^139^^,^^167^^,^^168^^,^^171^^,^^172^^,^^189^^,^^190^ Deán-Ben et al.^191^ employed spherical array PAT for real-time visualization of passage and trapping of individual B16 melanoma cells in the whole mouse brain. Imaging was performed with the laser wavelength and pulse repetition rate set to 700 nm and 50 Hz, respectively. About 100 frames of PA images were acquired before injection of B16 melanoma cells and averaged to determine a baseline. The system could identify and track B16 melanoma cells after injection by taking the difference between before and after images.^191^

#### 3.4.2 Human studies

**Fig. 9:**
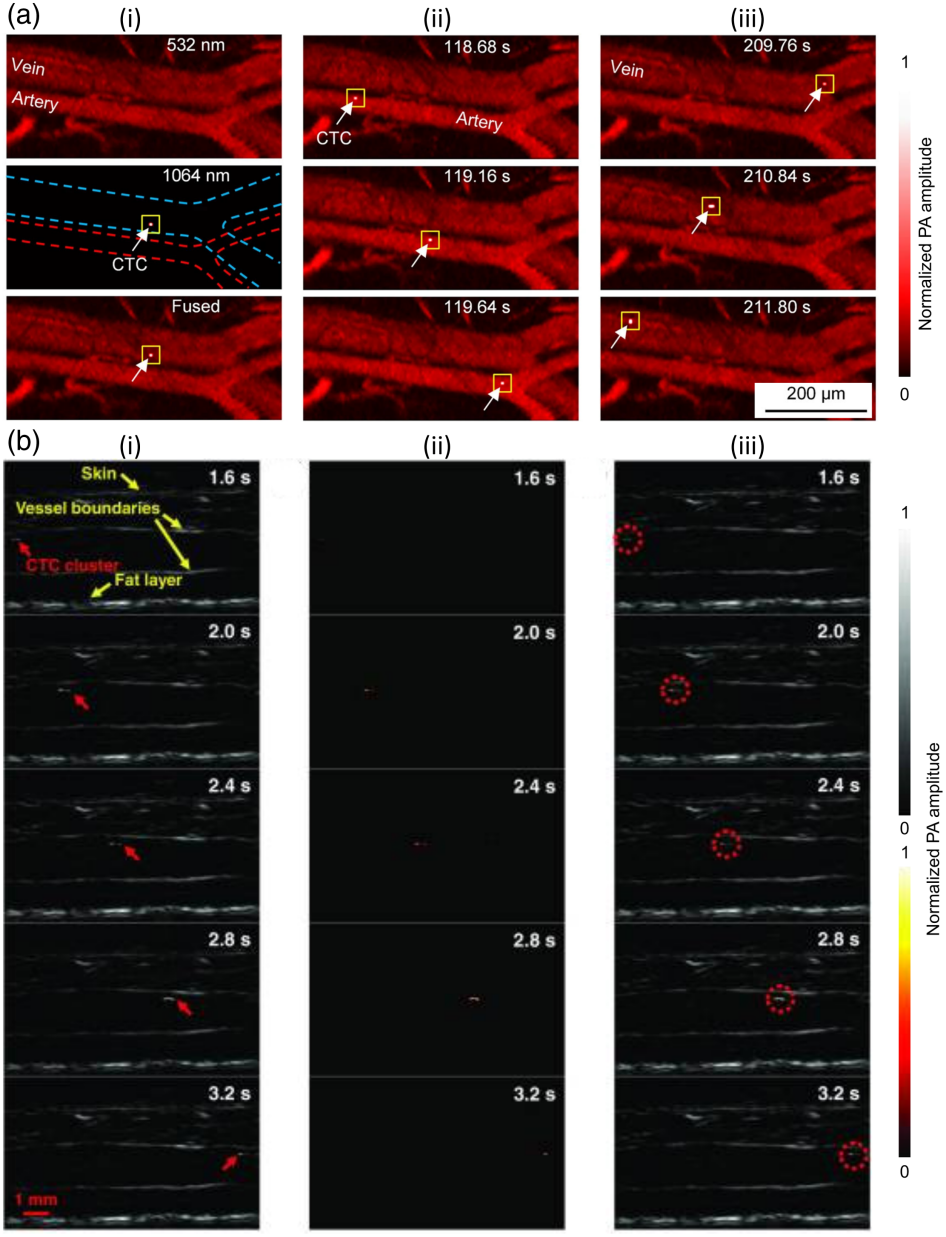
Imaging melanoma circulating tumor cells *in vivo*. (a) PA flow cytometry: channels shown individually and fused together (i), fused images showing movement of melanoma CTCs in an artery over time (ii), and in a vein (iii). (b) LA-PAT system uses differential analysis of images taken over time to find moving CTC cells: (i) PA snapshots of the melanoma CTC in the patient. The yellow arrows indicate structures, including the skin, vessel boundaries, and subcutaneous fat layer. The red arrows highlight the melanoma CTC. (ii) Differential PA images showing only the melanoma CTC. (iii) Differential PA images superimposed on structural images, highlighting the melanoma CTCs. Reproduced with permission from Ref. 192.

Galanzha et al.^166^ studied CTCs in healthy patients and patients with melanoma using PAFC *in vivo*. PAFC data from healthy patients were used to calculate false positives and to study PA artifacts.^166^ PAFC accurately identified unlabeled CTCs in 15/16 melanoma patients.^166^ The system uses MPAI with one channel for detection of blood and a second channel for melanin. The channels are shown individually and fused together in Fig. 9(a)(i), and fused images showing movement of melanoma CTCs in an artery [Fig. 9(a)(ii)] and a vein [Fig. 9(a)(iii)] are also presented. This system has relatively poor lateral resolution (Table 4), which the authors explain is caused by light beam blurring.^166^ Recently, PAT systems have also been used to detect CTCs. Hai et al.^171^^,^^192^ employed a LA-PAT system to detect melanoma CTCs in patients *in vivo*. Based on the optical absorption coefficient ratio, an excitation wavelength of 680 nm was chosen to maximize the contrast between melanoma CTCs and blood and achieve the highest detection sensitivity. Contrast-to-noise ratio (CNR) was used to quantify melanoma CTCs from the background tissue (RBCs). From the imaging session of the forearm of a positive patient with stage IV metastatic melanoma, a CTC was detected with a CNR of 9.4 [see Fig. 9(b)]. The single cells were captured at five frames and the flow speed was estimated to be 9.6  mm/s. They imagined 16 stage III and IV melanoma patients and successfully detected suspected melanoma CTCs in three patients. In fact, two of the three had disease progression, but four of those found CTC-negative also had disease progression. The lower rate of CTC detection by LA-PAT compared with PAFC could be attributed to the use of melanin as the single marker to identify and detect CTCs, and the fact that the patients were measured at only one clinical time point instead of on multiple repeat visits.^171^^,^^192^ Also, in the previous study, optical clearing techniques, including microdermabrasion and glycerol sonophoresis, increased PA signals by two- to threefold, due to the reduced light scattering in superficial skin layers.^166^ PAT has tremendous clinical potential in imaging CTCs and disease monitoring in melanoma, given the fact that PAT can image very deep in the tissue with very high optical contrast. Moreover, utilization of contrast agents for molecular PAI can further increase the sensitivity of PAT to imaging cancer cells that do not express melanin.

### 3.5 Virtual Histology

Recently, all-optical PA microscopy in reflection-mode has been shown to be able to form histology-like images of various cancers on unstained slides to distinguish tissue types.^142^^,^^267^ The principle behind this technology is that by concurrently measuring radiative and nonradiative (in the form of acoustic) emissions from tissue samples illuminated with light in the UV range (266 nm), it is possible to differentiate DNA, RNA, collagen, and elastin, among other chromophores. Nuclear contrast, in particular, comes from relaxation of DNA, and nonnuclear contrast from relation of extranuclear proteins. These enable high-resolution images remarkably analogous to traditional chemical haematoxylin and eosin (H&E) staining.^267^^,^^268^ This technique has very recently been applied to the analysis of skin biopsies suspected for melanoma.^269^^,^^270^

### 3.6 Exogenous Contrast Agents for Melanoma Detection

PAI most commonly utilizes intrinsic (endogenous) contrast agents, such as melanin and oxy- and deoxy hemoglobin to assess melanomas.^271^ However, an exogenous, melanoma-specific contrast agent could assist in assessing early-stage melanomas, tumor staging, surgical excision planning, detection of lymph node metastases, and detection of CTCs. In Table 5, some of these applications are demonstrated. Gold nanoparticles (AuNPs), due to their intrinsic bioinertness and highly tunable optical properties, have shown remarkable promise in cancer diagnostics and treatment.^273^ As a contrast agent, AuNPs display optical absorption and scattering cross-sections that are greater than those of organic dyes, making them appropriate contrast agents for several applications in biomedical optics, including PAI.

**Table 5:** Use of exogenous PA contrast agents for melanoma detection in mice.

PAI modality	Light source	US transducer	Resolution	Agent	Imaging model	Study result
AR-PAM	λ1: 778 nm	CF1: 50 MHz	LR: 45 to 506 μm	AuNC	Mice on the dorsal surface^173^	Volumetric images illustrate both tumors and blood vessels
	λ2: 570 nm	CF2: 10 MHz	AR: 15 to 150 μm			
		NE: 1				
LA-PAT	λ: 780 nm	CF: 21 MHz	AR: 75 μm	MAGE-Au-PFH-NPs	Mice, subcutaneous^175^	Tumor detection from enhanced PAI signal
		BW: 70%				
		NE: 256				
OR-PAM	λ1: 639 nm	CF: 3.5 MHz	NL	MNPs/Evans Blue dye	Mouse lymph nodes imaged *in vivo*^165^	Detection and treatment of metastases in SLN at a single cell level. Detection of micro-metastasis
	λ2: 850 nm	BW: NL				
		NE: 1				
PAFC	λ: 808 nm	CF: NL	NL	ID-HCuSNP, DOX and ICG	Mice, tail vein^195^	Cell membrane-camouflaged NPs have excellent self-recognition ability to the aimed tumor cells *in vivo*
		BW: NL				
PAFC	λ: 420 to 2300 nm	CF: 10 MHz	SR: 6 to 20 μm	AuNRs	Mice, tail vein^272^	Better absorption contrast introduced by AuNRs than control melanoma cells
		BW: NL				
		NE: 1				
PAFC	λ: 860 nm	CF: NL	NL	AuNPs in colony forming cells (ECFCs)	Mice, Tumor-bearing^196^	AuNP-loaded ECFCs generate higher PA signals than AuNPs alone
		BW: NL				

Note: λ, wavelength; CF, central frequency; NE, number of elements; BW, bandwidth; LR, lateral resolution; AR, axial resolution; SR, spatial resolution; NL, not listed; PAI, photoacoustic imaging; AR-PAM, acoustic resolution photoacoustic microscopy; LA-PAT, linear array photoacoustic tomography; AuNC, gold nanocrystals; MAGE-Au-PFH-NPs, Au nanorods and liquid perfluorocarbon (PFH) conjugated to a MAGE-1 antibody; ID-HCuSNP, hollow copper sulfide nanoparticle; DOX, doxorubicin; ICG, indocyanine green; PAFC, photoacoustic flow cytometry.

For example, gold nanorods (AuNRs) have been used to increase optical contrast in CTCs.^272^^,^^274^ Zharov et al.^272^ used PAFC to detect CTCs (human squamous carcinoma cell line SQ20B) labeled with AuNRs, which were 15  nm×52  nm in size and had a maximum absorption at 840 nm. The cancer cells labeled with AuNRs were injected into the mouse circulatory system through the tail vein. The real-time accumulation of AuNRs in the cells was monitored through an increase in PA signal. The maximum absorption contrast introduced by AuNRs compared with background tissue was ∼29∶1. The capability of AuNR was compared with that of a conventional contrast agent called ICG. At the same laser energy, linear PA signals from AuNR-labeled cancer cells were five to seven times stronger than the signals from cancer cells stained with ICG.

#### 3.6.1 Animal studies

**Fig. 10:**
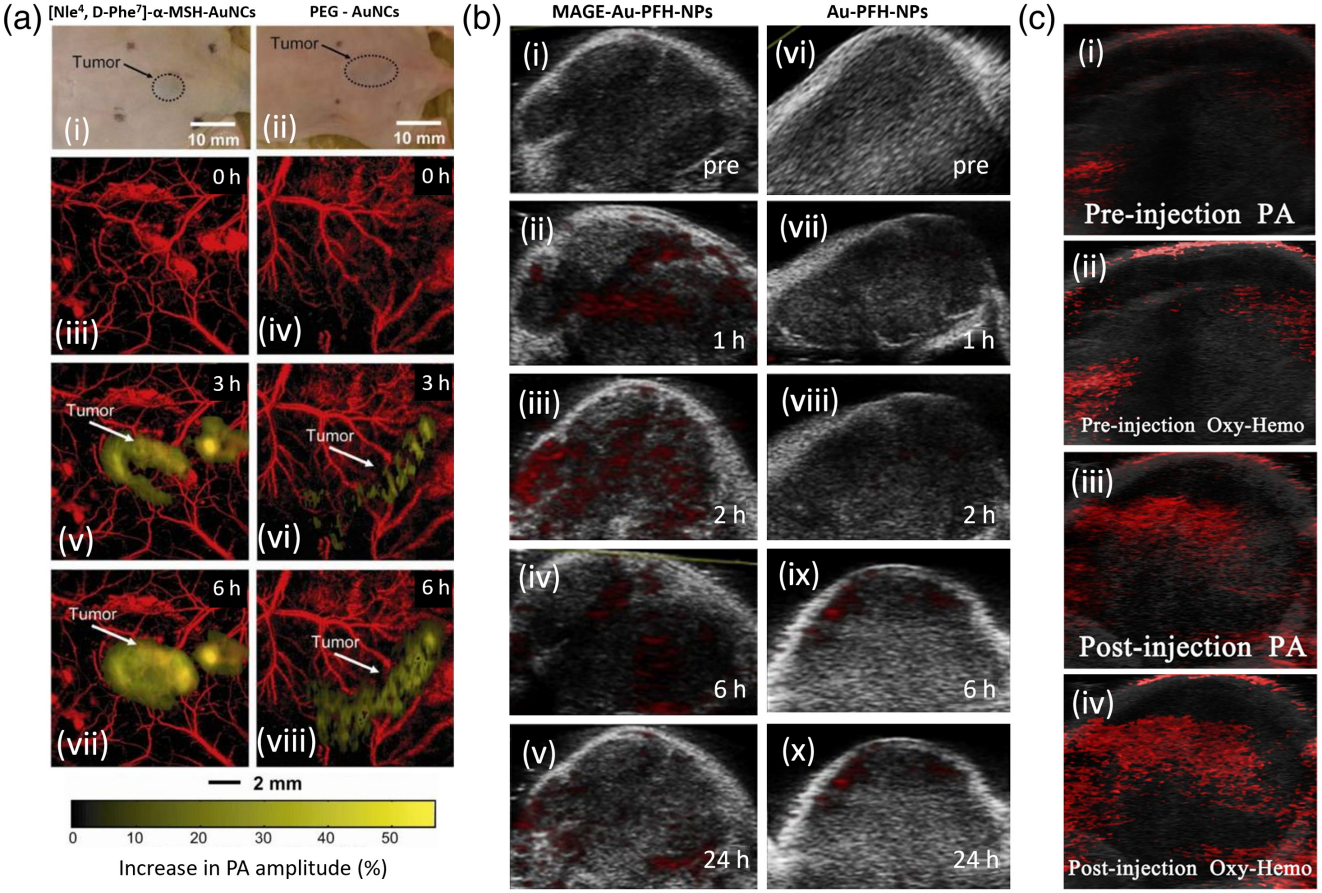
*In vivo* animal studies involving exogenous PAI contrast agents for melanoma detection. (a) *In vivo* MAP images of B16 melanomas using [Nle4,D-Phe7]-α-MSH- and PEG-AuNCs. (i), (ii) Photographs of tumor in mice before injection. (iii), (v), and (vii) Time course PA images after injection of  [Nle4, D-Phe7]-α-MSH-AuNCs. (iv) (vi), and (viii) Time course PA images after injection of PEG-AuNCs. Reproduced with permission from Ref. 173. (b) PA images of B16 melanomas in mice at different time points after the injection of (i) MAGE-Au-PFH-NPs and (ii) AU-PFH-NPs. Reproduced with permission from Ref. 175. (c) PA imaging of melanoma in mice (i) before and (ii) after the injection of ID-HCuSNP@B16F10 NPs. Reproduced from Ref. 195. MAP, maximum amplitude projection.

Kim et al.^173^ studied PA skin imaging of gold nanocrystals (AuNCs) bio-conjugated to $[{\mathrm{Nle}}^{4},D\text{-}{\mathrm{Phe}}^{7}]\text{-}\alpha$-melanocyte-stimulating hormone ($[{\mathrm{Nle}}^{4},D\text{-}{\mathrm{Phe}}^{7}]\text{-}\alpha \text{-}\mathrm{MSH}$) and compared them to poly(ethylene glycol) (PEG)-AuNCs. Melanoma cells strongly overexpress α-MSH receptors. In their study, mice were inoculated with B16 melanoma cells and either one or the other contrast agent was injected via tail vein. *In vitro* studies showed that melanoma cellular uptake of $[{\mathrm{Nle}}^{4},D\text{-}{\mathrm{Phe}}^{7}]\text{-}\alpha \text{-}\mathrm{MSH}\text{-}\mathrm{AuNCs}$ was ∼3.5 times greater than PEG-AuNCs uptake after 6- and 24-h incubation periods. *In vivo* experiments involved two groups of mice: one group (n=4) received $[{\mathrm{Nle}}^{4},D\text{-}{\mathrm{Phe}}^{7}]\text{-}\alpha \text{-}\mathrm{MSH}\text{-}\mathrm{AuNCs}$ and the other group (n=4) received PEG-AuNCs, both via tail vein [see Fig. 10(a)]. Six hours after injection, the PA signals of $[{\mathrm{Nle}}^{4},D\text{-}{\mathrm{Phe}}^{7}]\text{-}\alpha \text{-}\mathrm{MSH}$ AuNCs increased 38%, whereas the PEG-AuNCs increased only 13% [see Fig. 10(a)(iv)]. The number of AuNCs was quantified in the excised tumors using inductively coupled plasma mass spectrometry. The mean number of $[{\mathrm{Nle}}^{4},D\text{-}{\mathrm{Phe}}^{7}]\text{-}\alpha \text{-}\mathrm{MSH}$ AuNCs per gram of tumor was found to be 360% times greater than that of PEG-AuNCs, indicating the extremely high uptake of $[{\mathrm{Nle}}^{4},D\text{-}{\mathrm{Phe}}^{7}]\text{-}\alpha \text{-}\mathrm{MSH}$ by melanoma cells. More recently, Li et al.^175^ evaluated a nanoparticle composed of AuNRs and liquid perfluorocarbon (PFH) and conjugated it to a monoclonal antibody to melanoma-associated antigens (MAGE-1 antibody) and used an LA-PAT system for imaging. *In vitro* experiments showed a high number of MAGE-Au-PFH-NPs concentrated in the plasma membrane and cytoplasm of melanoma cells, compared with Au-PFH-NPs (control), which only showed a weak binding around tumor cells. Laser irradiation of nanoparticles with PFH results in a phase change (liquid PFH to gas microbubbles), increasing the acoustic impedance of surrounding tissues and subsequently causing a signal enhancement. *In vivo* PAI in tumor-bearing mice revealed a significantly higher PA signal intensity 2 h after MAGE-Au-PFH-NPs injection into the tail vein compared to Au-PFH-NPs; higher conjugate concentrations produced greater PA signals [see Fig. 10(b)].

Wu et al.^195^ developed cell membrane-camouflaging hollow copper sulfide nanoparticles (ID-HCuSNP) by coating the membrane of melanoma cells with doxorubicin and ICG-loaded hollow copper sulfide NPs to enhance their targeting ability. They injected a mouse model with ID-HCuSNP and after 4 h, and they observed a strong local PA signal in the tumor area due to the accumulated NPs.^195^ This showed cancer cell membrane-camouflaged NPs having an excellent self-recognition ability to the aimed tumor cells *in vivo* [see Fig. 10(c)]. Galanzha et al.^165^ used PAT with mapping techniques (multicolor PA lymph flow cytometry, PA lymphography, and absorption image cytometry) to examine metastasis to the prenodal lymph vessels and SLNs in mice *ex vivo* and *in vivo*. Specifically, after a visible primary melanoma tumor had developed in the mouse ear, they injected magnetic nanoparticles (MNPs) that provided stronger PA signals from lymphatics and SLNs at 639 nm. However, similar NIR absorption spectra of MNPs and melanin made it difficult for spectral identification of melanoma metastasis in the presence of MNPs. To resolve this problem, they used Evans Blue (EB) dye, which absorbs poorly at 639 nm only, while the presence of melanoma cells in lymphatics and in a node was identified through the appearance of PA signals at 850 nm. Because absorption of melanin also occurred at 639 nm above the background from EB dye, they used specific ratio of PA signals at 639 and 850 nm for additional identification of melanoma cells. They injected EB dye near melanomas derived from B16F10 cells and imaged the region in week 1 and 2 postinoculation. The number of metastatic melanoma cells in transit increased from 0.26  cells/min in week 1 postinoculation to 2.13  cells/min in week 2 postinoculation. The percentage of PA signal covering the examined SLN area increased from 6% in week 1 postinoculation to 39% in week 2 postinoculation. In the histology results, lymph nodes showed no metastases after 1-week inoculation and single metastases after 2 weeks inoculation. Thus the PA system was able to detect early micrometastases that could not be visualized in histology, illustrating its ability to detect even single metastatic cells (at week 1 postinoculation).

Despite the number of reports on the successful demonstration of Au nanomaterials for cancer theranostics,^275^^,^^276^ accumulations in the liver and spleen due to resident macrophages that form the mononuclear phagocyte system are still an issue.^277^^,^^278^ Armanetti et al.^196^ utilized endothelial colony forming cells (ECFCs) to carry AuNPs and explored the antitumor effects and the tumor-homing efficiency following single intravenous injection into tumor-bearing mice. They assessed AuNPs biodistribution in freshly excised mice organs at different time points post administration by exploiting the PA properties of AuNP-enriched ECFCs. They demonstrated *in vitro* that AuNP-loaded ECFCs are able to generate higher PA signals than AuNPs alone and also display spectral fingerprints that enable a reliable detection of labeled cells following intravenous injection.^196^

Toxicity is always a concern when developing exogenous contrast agents. Factors for assessing biocompatibility include inertness, metabolism, and effective clearance rates. Material composition, surface modifications, shape, and size must all be designed carefully to maximize biocompatibility. The growing field of PAI for cancer diagnosis and treatment planning is accelerating research in biocompatible exogenous contrast agents.^279^^,^^280^

## 4 Discussion

The scalability of PAI technology (from OR-PAM to AR-PAM to PAT) allows a wide range of applications including melanoma detection and depth determination, tumor-related angiogenesis, lymph node metastases, and presence of CTCs, all either with endogenous or exogenous contrast agents. Although these applications have been explored using small animal models for a couple dozen years, in the past decade or so, human observational studies have demonstrated the clinical potential for these systems. Although these technologies need more confirmational human studies, they lay a strong foundation for the near-term translation of PAI systems to the clinic for melanoma disease detection and management.

In terms of initial diagnosis and melanoma depth determination, if melanoma is in its very earliest stages (at the epidermis), OR-PAM provides rich information on tumor presence and angiogenesis—therefore, it can be used not only for detection but for accurate treatment planning, as the extent of angiogenesis may predict tumor aggressiveness. AR-PAM could be a better choice for deeper melanomas (approximately ≥1  mm). Although PAT could be implemented using ultrahigh frequency transducers, the use of it to detect and characterize early stage melanomas has not been explored, perhaps due to the requirement for a much-more sophisticated data acquisition system, transducer focal length, and complex light illumination configuration. Another shortcoming of PAT is that it is limited to providing 2D images, unless it is implemented with a scanning system, which adds complexity. Nevertheless, tunable lasers with a wide range of wavelengths (to accurately distinguish melanoma biomarkers such as melanin) are much more accessible for PAT implementation compared to AR/OR-PAM. Therefore, where permitted, high or ultrahigh frequency PAT could be a good choice for melanoma detection and depth determination. Complementing current histopathology of excised tissue, recently, all-optical PA microscopy in reflection-mode has been shown to form histology-like images of various cancers on unstained slides to distinguish tissue types. This technique has great potential to be in the clinic for rapid dermatological tissue analysis. Once a melanoma is appropriately detected, knowing whether it has metastasized to the SLN is the next important question for treatment planning. Because of the depth of SLNs, PAT might be the best choice, and in fact, in Table 3, all of the human studies of SLNs were performed by PAT, although in some cases, AR-PAM could potentially be used as well. In cases where melanoma is confirmed metastasized to the SLN (or beyond), detection of CTCs is very valuable for monitoring responses to cancer treatment and evaluating prognosis. With PAI being a technology based on high-energy laser, fast switching between imaging and ablation of CTCs has been investigated in small animals, it could be translated into a theranostic application, once the concept is sufficiently confirmed through clinical studies.

Although melanin is an adequate optical contrast for early-stage melanomas, it is not sufficient for later stage melanomas due to the limitation of light penetration in deep structures. Exogenous contrast agents have been shown to increase PA signals beyond the signal level obtained using the label-free PAI (endogenous contrast agents). As discussed in Sec. 3.5, different tumor cell targets (antigens) of the antibody-coupled exogenous contrast agents have been tested on animal models. While the results seemed promising, exogenous contrast agents need to be proven safe to have clinical utility. In addition, before exogenous contrast agents are studied in humans, a unique target protein present on melanoma cells and absent on healthy cells will need to be identified, such as galectin-3 or collagen XVII, which have recently been shown to be overexpressed in melanoma cells.^281^ Furthermore, few if any studies have investigated whether a contrast agent could be applied topically on the melanoma lesion as opposed to intravenously, which could mitigate potential toxicity. However, PAI will likely need to gain clinical utility before exogenous contrast agents are rigorously investigated in humans.

While PAI holds great clinical potential, safety and cost are factors that may limit the ease of clinical translatability. There is a safety concern about tissue damage from the light excitation. The American National Standards Institute has already defined the maximum permissible exposure, which is the level of electromagnetic radiation that a person can be exposed to without harmful effects, for skin imaging;^91^ another is potential damage to the eyes of the patient or the physician from stray laser light, which can be mitigated by using appropriate protective eyewear. Cost is a further potential limitation that can be mitigated by mass production of PAI systems. A final hurdle is the need for pulsed nanosecond lasers, which can be costly. Low-cost laser sources such as laser diodes and light-emitting diodes (LEDs) have been shown to be effective in some PAI applications.^282^^–^^284^

In recent years, there has been a growing interest among various companies in the development and commercialization of PAI systems for medical applications.^145^^,^^285^ Among them are iThera Medical GmbH,^105^^,^^130^^,^^180^^,^^286^^–^^297^ FUJIFILM VisualSonics,^135^^,^^171^^,^^187^^,^^298^ ENDRA Life Sciences Inc.,^299^^–^^303^ TomoWave Laboratories,^304^^–^^307^ and Seno Medical Instruments.^308^^,^^309^ Although there exist commercial systems for skin imaging, there has not been any customized clinical system for melanoma imaging applications.

## 5 Conclusions

Prior CM studies in animals and humans have illustrated PAI’s potential use in identifying tumor depth, analysis of angiogenesis, detection of lymph node metastasis, detection and ablation of CTCs, and ability to generate histology-like images of various cancers on unstained slides. These applications stem from the high spatial and temporal resolution, adequate penetration depth, and label-free nature of this imaging modality.

Many of these opportunities are based on the use of melanin as a high-absorbing chromophore. However, reliance on melanin has a few limitations. First, as noted above,^251^ for tumors with very high density of melanoma, it can be difficult for PAI to penetrate through the tumor to detect lower boundaries without assistance from, for example, US structural information. Second, amelanotic melanoma, which accounts for 1% to 8% of all melanomas, is a form of melanoma with little to no pigment (lesion pigment not differentiated from skin).^310^ Methods focused on melanin detection could miss these lesions, and PAFC likely will miss amelanotic CTCs.

Despite the advantages of PAI described in this review, this technique has not yet been used as a diagnostic-assistant tool in large-scale clinical studies. One possible way to enroll large groups of patients in future studies would be to incorporate PAI before performing procedures to remove lesions suspicious for melanoma or before lymph node excisions. This would allow for characterization of PAI images from various pigmented lesions that may clinically resemble melanoma, such as benign nevi, dysplastic nevi, lentigo, etc., as well potential malignant lymph nodes. As more images are collected and analyzed, algorithms on the recognition of melanoma in PA images will mature.

Safety and cost are two key parameters in clinical translatability of PAI. The use of laser light must be limited to the maximum permissible levels for skin imaging, and protective eyewear or confining apparatus can mitigate potential damage to eyes. PAI system costs can be reduced by using a combination of LEDs and development of low-cost US transducers designed specifically for PA applications.

## Data Availability

The data presented in this review article has been collected from different sources and each source has been cited in the article for further details.
